# Tumor-Derived Lactate Drives Malignant Progression of Refractory Papillary Thyroid Carcinoma via the H3K18la-STAT1-LDHA Axis

**DOI:** 10.7150/ijbs.120277

**Published:** 2025-10-01

**Authors:** Zheng Zhou, Chao He, Xumeng Wang, Xinguang Jin, Liping Wen, Yan Yang, Quan Zhou, Weibin Wang, Lisong Teng

**Affiliations:** 1 Department of Surgical Oncology, The First Affiliated Hospital, Zhejiang University School of Medicine, Hangzhou, China.; 2 Institute of Immunology, Zhejiang University School of Medicine, Hangzhou, Zhejiang, China.

## Abstract

Papillary thyroid carcinoma (PTC) remains among the most prevalent endocrine malignancies globally, with its incidence steadily rising. Although clinical outcomes are generally favorable, a clinically significant subset of patients exhibits highly aggressive tumor phenotypes, characterized by larger tumor size and increased lymph node metastasis. Accumulating evidence implicates metabolic reprogramming and epigenetic dysregulation as pivotal drivers of tumor progression. Lactate, one of the byproducts of tumor metabolism, has recently garnered attention for its regulatory functions beyond metabolism. Histone lactylation, a recently identified epigenetic modification dynamically regulated by intracellular lactate accumulation, has emerged as an important regulator of tumor proliferation, metastasis, immune evasion, and therapeutic resistance. However, the functional implications and mechanistic underpinnings of histone lactylation in PTC remain largely unexplored.

Here, we report significantly elevated pan-lysine lactylation and histone H3 lysine 18 lactylation (H3K18la) levels in clinical PTC specimens, with tumor tissues exhibiting markedly higher levels compared to adjacent normal thyroid tissues., correlating positively with aggressive clinicopathological features. Relevant cellular phenotypic assays further support this conclusion. Mechanistically, we demonstrate that H3K18la modification directly facilitates the transcriptional activation of Signal Transducer and Activator of Transcription 1 (STAT1). Activated STAT1 subsequently promotes transcriptional upregulation of Lactate Dehydrogenase A (LDHA), thereby enhancing lactate biosynthesis and establishing a self-perpetuating positive feedback loop. Consequently, tumor-derived lactate orchestrates and sustains malignant progression in PTC through this “H3K18la-STAT1-LDHA” regulatory axis.

Collectively, our findings uncover a novel mechanistic linkage between tumor metabolism and epigenetic regulation in PTC, providing critical insights into thyroid cancer pathogenesis. Furthermore, therapeutic targeting of the H3K18la-STAT1-LDHA axis may represent an innovative and promising strategy to improve outcomes for patients with aggressive and refractory PTC.

## Introduction

Thyroid cancer is among the most common endocrine malignancies worldwide, with its incidence rising dramatically by approximately 313% over the past four decades, reflecting a significant global epidemiological shift. Recent GLOBOCAN data indicate that papillary thyroid carcinoma (PTC), the most prevalent histological subtype, exhibits an age-standardized global incidence rate of approximately 3.0%, which rises markedly to 4.9% in women, ranking fifth among all female cancers 1. In China, epidemiological statistics from 2022 revealed approximately 466,100 new thyroid cancer cases, positioning it as the third most frequently diagnosed malignancy nationwide 2. Although PTC is generally characterized by an indolent course and favorable prognosis, with surgical resection alone often resulting in high cure rates 3, recurrence, lymph node metastasis, and distant metastasis occur in about 5%-21% of patients, significantly impacting their long-term survival. Additionally, a subset of PTC patients presents with aggressive clinical manifestations at diagnosis, such as tracheal invasion or recurrent laryngeal nerve involvement 4, 5. In these high-risk or refractory cases, standard surgical management alone is often insufficient, necessitating individualized treatment approaches. Such approaches frequently involve multitarget tyrosine kinase inhibitors and mutation-specific targeted therapies, including BRAF/MEK inhibitors (dabrafenib/trametinib) or RET inhibitors (selpercatinib, pralsetinib), to achieve improved clinical responses and disease control 3. Thus, comprehensively elucidating the genetic alterations and molecular regulatory mechanisms underlying PTC initiation and progression is critical for effective risk stratification, therapeutic innovation, and the identification of reliable diagnostic and prognostic biomarkers.

A growing body of evidence indicates that the malignant behavior of PTC, as a prototypical endocrine tumor, is intricately associated with dysregulated cellular metabolism and epigenetic reprogramming 6. As tumor metabolism research increasingly intersects with epigenetics, metabolic intermediates have emerged not merely as substrates for energy generation and biosynthesis but also as pivotal modulators of chromatin structure, gene transcription, and cellular fate determination 7, 8. Beyond their traditional metabolic functions, these metabolites also influence multiple oncogenic signaling pathways, thereby contributing to tumor initiation, progression, and therapy resistance 9.

Under physiological conditions, glucose is predominantly metabolized through oxidative phosphorylation within the tricarboxylic acid cycle to generate adenosine triphosphate. In contrast, cancer cells preferentially employ aerobic glycolysis, even under sufficient oxygen conditions, to rapidly produce lactate—a metabolic phenomenon known as the “Warburg effect” 10. Notably, Zhao et al. (2019) first identified that lactate can be enzymatically converted into lactyl-CoA, which subsequently serves as a substrate for histone lysine lactylation catalyzed by the histone acetyltransferase EP300 11. This newly characterized epigenetic modification—histone lactylation—facilitates chromatin relaxation and transcriptional activation, thereby redefining lactate as a bioactive signaling molecule and establishing a critical link between altered metabolism and epigenetic regulation.

Among identified histone lactylation sites, histone H3 lysine 18 lactylation (H3K18la) has emerged as one of the most prominent and functionally significant marks. Accumulating studies have demonstrated elevated H3K18la expression in various solid tumors, including non-small cell lung cancer, colorectal cancer, and bladder cancer, and others, correlating closely with malignant phenotypes such as enhanced proliferation, metastasis, immune evasion, and treatment resistance 6, 12-17. Nevertheless, the functional role of histone lactylation in PTC remains largely unexplored, and thus far, this epigenetic modification has not been successfully targeted therapeutically to reprogram metabolic pathways in PTC. Furthermore, the detailed regulatory networks and underlying molecular mechanisms controlling histone lactylation in this context remain unclear. Therefore, a systematic investigation of the biological significance and downstream signaling events mediated by histone lactylation, particularly H3K18la, could provide novel mechanistic insights and identify promising therapeutic targets for precision oncology in PTC.

The current study aims to clarify the functional implications of histone lactylation—particularly H3K18la—in the tumorigenesis and progression of PTC and to elucidate its underlying molecular mechanisms. Our findings demonstrate that lactate accumulation resulting from altered tumor metabolism significantly increases H3K18la levels in both PTC tissues and cellular models. Elevated H3K18la expression correlates positively with aggressive tumor characteristics and unfavorable clinicopathological parameters. Mechanistically, lactate-induced elevation of H3K18la promotes the transcriptional activation of Signal Transducer and Activator of Transcription 1 (STAT1). Further analyses revealed that STAT1 directly binds to the promoter region of Lactate Dehydrogenase A (LDHA), enhancing its transcription and thus promoting increased lactate production. Collectively, this establishes a tumor-derived lactate-driven positive feedback loop, defined herein as the “H3K18la-STAT1-LDHA” axis.

In conclusion, this study provides the first comprehensive evidence highlighting the critical biological role and mechanistic significance of histone lactylation, particularly H3K18la, in PTC. We propose that tumor-derived lactate orchestrates malignant behaviors through the “H3K18la-STAT1-LDHA” regulatory axis. Therapeutic targeting of this pathway may offer a novel and promising approach, complementing existing targeted therapies, particularly for patients with recurrent or treatment-refractory PTC.

## Materials and Methods

### Patients and Specimens

This study enrolled 178 patients diagnosed with PTC who underwent surgical resection at the Department of Surgical Oncology, the First Affiliated Hospital of Zhejiang University School of Medicine, between March 1, 2022, and December 1, 2022. Tissue microarray (TMA) was constructed from formalin-fixed, paraffin-embedded tumor samples for subsequent immunohistochemical (IHC) analysis. All patients provided informed consent regarding the potential scientific use of their specimens. Comprehensive clinicopathological data, including surgical records, pathological staging (tumor-node-metastasis, TNM), and follow-up survival information, were collected. Ethical approval with a waiver of informed consent was granted by the Clinical Research Ethics Committee of the First Affiliated Hospital, Zhejiang University School of Medicine (Approval No.: [IIT20250579B]).

### Cell Lines and Cell Culture

Human PTC cell lines (TPC1 and IHH4) and the normal thyroid follicular epithelial cell line Nthy-ori 3-1 were obtained from the American Type Culture Collection (ATCC, Manassas, VA, USA). Cells were cultured in RPMI-1640 medium (VivaCell, C3010-0500) supplemented with 10% fetal bovine serum (FBS; HXBio, HX-FBS-01), 100 μg/mL streptomycin, and 100 IU/mL penicillin (Yeasen Bio., 60162ES), maintained at 37 °C in a humidified incubator with 5% CO₂. Cell identities were authenticated via short tandem repeat (STR) profiling prior to use.

### Lentiviral Transduction and Stable Cell Line Establishment

Lentiviral vectors and overexpression plasmids were synthesized by General Biology Co., Ltd. (Anhui, China). PTC cells were seeded at 2.5 × 10⁵ cells/well in six-well plates, cultured for 24 hours, and then transduced with lentiviral constructs or empty vector controls. Stable transfectants were selected with 2.5 μg/mL puromycin over a period of two weeks. Established stable cell lines were expanded, harvested, and cryopreserved for subsequent experiments.

### Glucose Uptake and L-Lactate Production Assays

Glucose consumption and L-lactate production were quantified using commercial assay kits (Glucose Assay Kit based on hexokinase method and Lactic Acid Assay Kit, Nanjing Jiancheng Bioengineering Institute, A019-2-1 and A154-2-1; Nanjing, China) according to the manufacturers' protocols.

### Cell Counting Kit-8 (CCK-8) Assay

Cells were seeded into 96-well plates at a density of 3,000 cells/well in 100 μL complete medium, with triplicate wells per condition. After 24-hour incubation, 10 μL CCK-8 reagent (APExBIO, K1018) was added at indicated time points (0, 24, 48, and 72 hours). Following further incubation at 37°C for 2 hours, absorbance at 450 nm was measured using a microplate reader. Cell growth curves were plotted, and proliferation rates were calculated accordingly.

### Cell Invasion Assay

Cell invasion was evaluated using 24-well Transwell chambers (Corning, #3422). Briefly, 1 × 10⁴ cells in 200 μL serum-free medium were seeded into the upper chamber inserts, while 700 μL complete medium was added to the lower chambers. After 24 hours, invaded cells were fixed with 4% paraformaldehyde, stained with 0.05% crystal violet, and non-invaded cells were removed gently with cotton swabs. Invaded cells were counted in three random fields per sample under a microscope (20× magnification).

### Wound Healing Assay

Cells were seeded into six-well plates (5 × 10⁵ cells/well) in triplicate and cultured to confluence. Monolayers were scratched vertically using a sterile 10-μL pipette tip, followed by gentle washing with phosphate-buffered saline (PBS). Initial scratch widths were photographed immediately. Cells were cultured in serum-free medium, and wound closure was monitored at identical locations every 24 hours. Wound closure rates were calculated based on measurements from captured images. Closure rates were quantified from captured images by measuring linear distances (pt) using Adobe AI.

### RNA Extraction and RT-qPCR Analysis

Total RNA was extracted using an RNA Purification Kit (EZBioscience, B0004DP). One microgram RNA was reverse-transcribed using Evo M-MLV Reverse Transcriptase (Accurate Biology, AG11707). Quantitative PCR was performed with Universal SYBR Green Fast qPCR Mix (ABclonal, RK21203) on a CFX96 Real-Time PCR Detection System (Bio-Rad, StepOnePlus). Gene expression levels were normalized to β-actin and calculated using the 2^^-∆∆Ct^ method. Primer sequences are listed in Table 1.

### Western Blot Analysis

Protein lysates were obtained using RIPA buffer (Beyotime, P0013B) with protease and phosphatase inhibitors (1:100 dilution). Proteins were quantified and subjected to SDS-PAGE, transferred onto PVDF membranes (Millipore, USA), and blocked with QuickBlock Blocking Buffer (Beyotime, P0252). Membranes were incubated overnight at 4 °C with primary antibodies and subsequently with HRP-conjugated secondary antibodies for 1 hour at room temperature. Bands were visualized using enhanced chemiluminescence reagent (Yeasen Bio., 36208ES60). β-actin served as a loading control. Antibodies used are detailed in Table 2.

### Immunohistochemistry

Tissues were fixed in 4% paraformaldehyde, embedded in paraffin, sectioned, stained, and examined following institutional pathology guidelines. Immunohistochemical staining was analyzed using Visiopharm software. H-score was calculated as follows:

H-score = (weak positive %) × 1 + (moderate positive %) × 2 + (strong positive %) × 3

### Cleavage Under Targets and Tagmentation (CUT&Tag) Assay

CUT&Tag assays were performed with the CUT&Tag Assay Kit (Abclonal, RK20265). Briefly, cells were bound to concanavalin A-coated magnetic beads, permeabilized with digitonin, and sequentially incubated with primary antibodies (anti-H3K18la), secondary antibodies, and hyperactive Protein A - Protein G - Micrococcal Nuclease (pAG/MNase) fusion protein. DNA fragments were amplified with Illumina adapters, and libraries passing quality control (concentration >2 ng/µL) were sequenced at the Analysis Center of Agrobiology and Environmental Sciences, Zhejiang University.

### Cleavage Under Targets and Release Using Nuclease (CUT&RUN) Assay

CUT&RUN assays followed previously described protocols (Meers et al.) 18 using the CUT&RUN Assay Kit (CST, #86652). Cells bound to ConA beads were incubated overnight at 4°C with anti-H3K18la antibody, followed by targeted chromatin cleavage with pAG/MNase. Released DNA was purified (CST, #14029) and analyzed by qPCR (SimpleChIP® Universal qPCR Master Mix, CST, #88989).

### Dual-Luciferase Reporter Assay

The STAT1 promoter region (-2000 to 0 bp) was cloned into pGL3-basic (General Biology Co., Ltd.). Cells co-transfected with pGL3-STAT1 or empty vector plus pRL-TK (Yeasen Bio., 11402ES60) were incubated ± Nala/2-DG for 48 h. Luciferase activity was measured, normalized to Renilla luciferase.

### Subcutaneous Xenograft Model in Nude Mice

IHH4 cells (7.5×10⁶) were subcutaneously injected into nude mice. Upon reaching 150 mm³, mice received intraperitoneal injections of 2-deoxy-D-glucose (2-DG, 50 mg/kg) and sodium lactate (Nala, 1 mg/kg) every other day. Tumor volumes were monitored, mice euthanized at day 16, and tumors weighed. Experiments complied with institutional guidelines (Approval No.: [2025095]).

### Acquisition and processing of single-cell data

Single-cell RNA sequencing data from primary PTC and para-tumor tissues (GEO: GSE184362) were processed following standard workflows. Gene expression matrices were globally normalized (scaling factor = 10,000) and log-transformed [log (1 + x)] using the “*NormalizeData”* function in “**Seurat”**. Batch effects were corrected with “**harmony”**, and unsupervised clustering was performed with *FindClusters* (resolution = 0.5). Louvain clusters were visualized via t-SNE, and cell types were annotated manually according to Wang *et al.* 19. Thyroid tissue subpopulations were identified, and differential expression analysis was conducted using “**DESeq2”**.

### Statistical Analysis

Data are presented as mean ± standard deviation. Student's *t*-test was used for comparisons. Statistical analysis was performed using R software (version 4.3.3). *P* < 0.05 indicated significance.

## Results

### Histone Lactylation Is Elevated in PTC and Correlates with Aggressive Tumor Features

**Figure 1:**
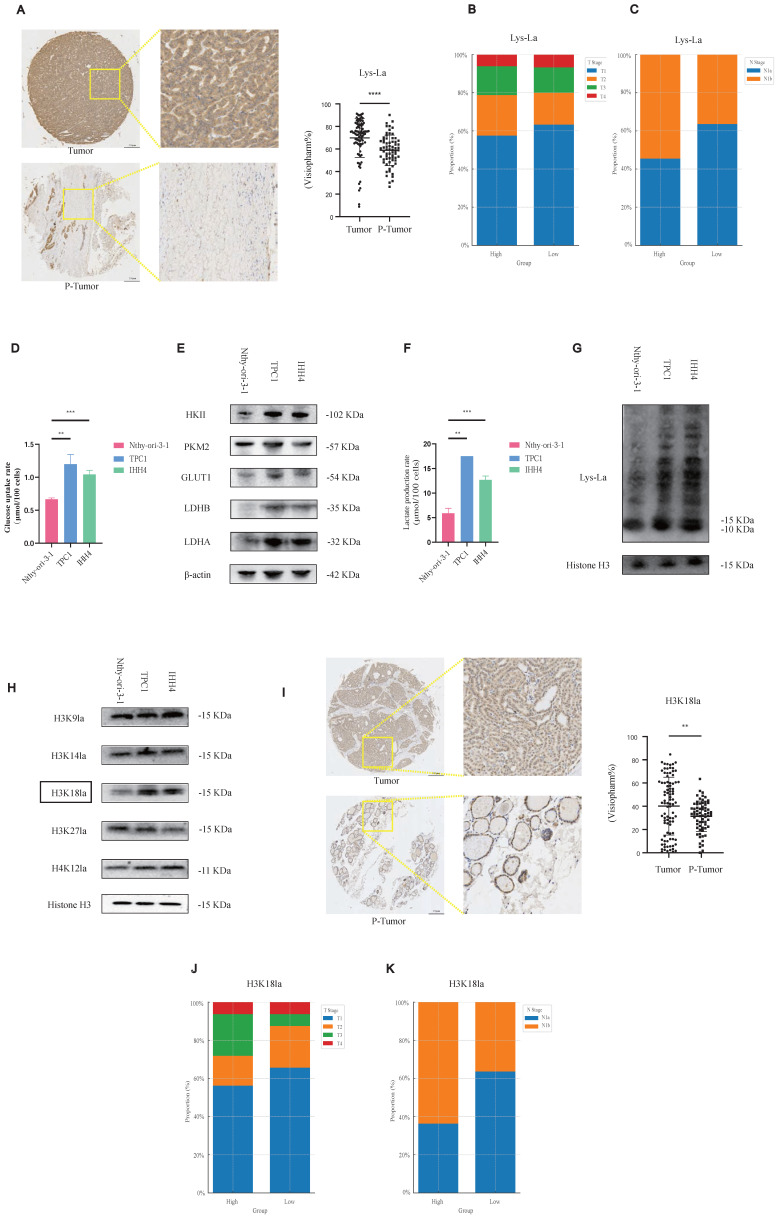
Histone lactylation is elevated in PTC, with H3K18la being the most prominent site, and correlates with aggressive tumor phenotypes. **(A)** IHC staining of Lactylation in TMA from PTC patients. **(B-C)** Lactylation IHC staining in PTC samples stratified by T stage (B) and N stage (C). **(D)** Glucose uptake rate in PTC cell lines compared with normal thyroid epithelial cells. **(E)** Protein levels of key glycolytic enzymes in PTC cell lines versus normal controls. **(F)** L-lactate production rates in PTC versus normal thyroid cell lines. **(G)** Lactylation levels in PTC versus normal thyroid cell lines. **(H)** Expression of different histone lactylation marks in PTC versus normal thyroid cell lines, highlighting H3K18la. **(I)** IHC staining of H3K18la in TMA samples from PTC patients. **(J-K)** H3K18la expression stratified by T stage (J) and N stage (K) in PTC patients.

Our TMA analysis revealed significantly elevated pan-lysine lactylation levels in PTC tumor tissues compared to para-tumor tissues (p-tumor, Figure 1A; representative IHC staining in Figure S1A).

Considering variations in tumor (T) and nodal (N) staging among patients, we stratified cases into high- and low-lactylation groups based on staining intensity. Patients exhibiting high lactylation levels frequently presented with advanced T stage and lateral cervical lymph node metastasis (Figures 1B and 1C).

Our results suggest that lactylation may play a pro-tumorigenic role in PTC, we next sought to further investigate this possibility at the cellular level. We assessed glycolytic activity in PTC cell lines compared to normal thyroid epithelial cells. PTC cells demonstrated increased glycolytic rates (Figure 1D), elevated protein levels of key glycolytic enzymes such as Hexokinase 2 (Figure 1E), higher L-lactate production (Figure 1F), and consequently enhanced histone lactylation (Figure 1G).

To further elucidate specific lactylation marks potentially involved in PTC progression, we performed a targeted screen. Among candidate modifications, H3K18 lactylation (H3K18la) was notably elevated in PTC cells relative to normal controls (Figure 1H). TMA-based IHC analysis confirmed significantly higher H3K18la expression in tumor versus p-tumor tissues (Figure 1I), with elevated H3K18la correlating with advanced T stage and cervical lymph node metastasis (Figures 1J and 1K; Figure S1B). Moreover, pan-lysine lactylation levels were positively correlated with H3K18la levels (Figure S1C). In addition, we examined other potential histone lactylation sites, such as H3K9la and H4K12la, as well as other histone modifications (H3K18ac and H3K18cr); however, no statistically significant differences were observed, and other lactylation sites appeared to exert little influence on the malignant behavior of PTC. In fact, other histone modifications even showed a paradoxical trend, whereby lower modification levels were associated with enhanced malignant progression. This paradox may reflect complex epigenetic crosstalk in PTC biology (Figure S1D-S1I, Figure S2A-S2F).

Collectively, these data indicate that histone lactylation, particularly H3K18la, may contribute to malignant progression in PTC.

### Histone Lactylation Regulates PTC Cell Proliferation and Invasion

**Figure 2:**
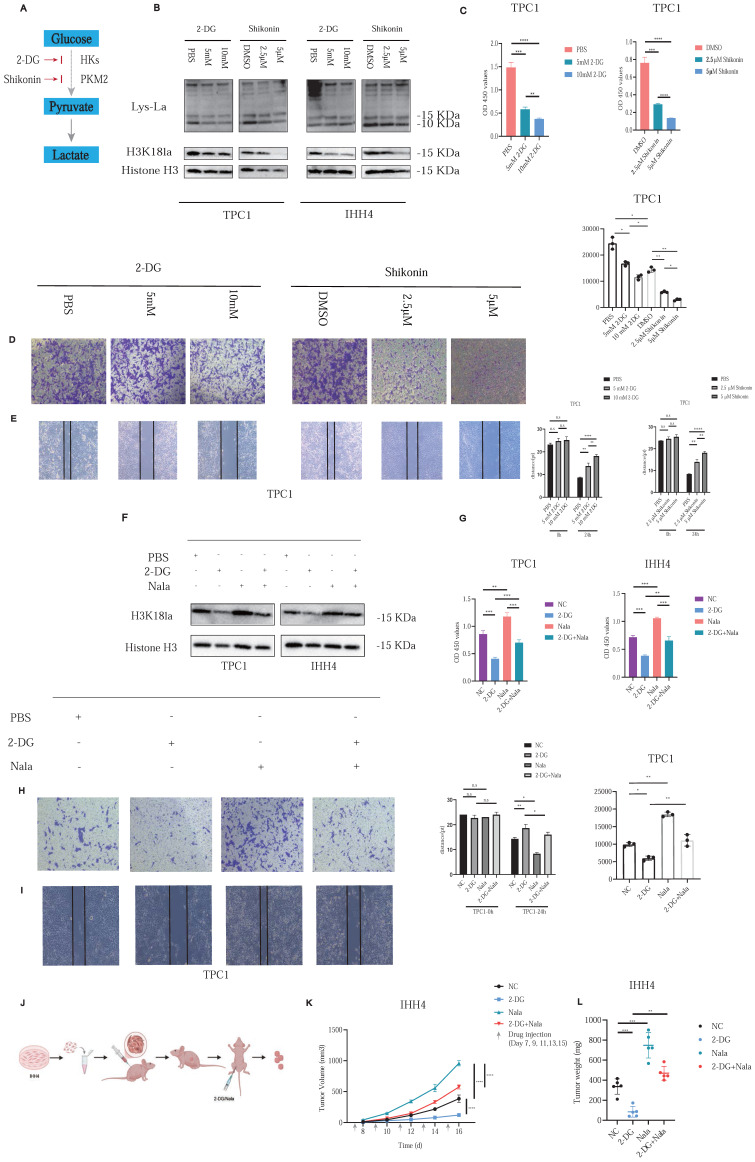
Histone lactylation modulates PTC cell proliferation and invasion. **(A)** Schematic of glycolysis pathway and inhibitors: Shikonin suppresses PKM2 activity, and 2-DG inhibits the conversion of glucose to glucose-6-phosphate. **(B)** Dose-dependent effects of 2-DG and Shikonin on Lactylation and H3K18la levels in PTC cells. **(C)** Inhibition of PTC cell proliferation by 2-DG and Shikonin. **(D-E)** Inhibition of PTC cell invasion by 2-DG and Shikonin. **(F)** Rescue of H3K18la levels by Nala under 2-DG treatment. **(G)** Partial recovery of cell proliferation by Nala following 2-DG treatment. **(H-I)** Partial restoration of invasion ability by Nala under 2-DG treatment. **(J)** Schematic of xenograft tumor modeling and intraperitoneal injection in nude mice. **(K-L)** Tumor growth curves (K) and tumor weight comparisons (L) in mice treated with different regimens.

To investigate the functional role of histone lactylation in PTC malignancy, we focused on H3K18la, identified previously as a critical lactylation site. 2-DG, blocking glucose-6-phosphate formation, and Shikonin (A12125, AdooQ), inhibiting Pyruvate Kinase M2 (PKM2), were employed to reduce glycolytic flux and subsequent histone lactylation (Figure 2A).

Treatment of PTC cell lines with increasing concentrations of 2-DG or Shikonin resulted in dose-dependent decreases in histone lactylation and H3K18la specifically (Figure 2B). Correspondingly, cell proliferation (Figure 2C; Figure S3A) and invasive capacity (Figures 2D and 2E; Figures S3B and S3C) were significantly inhibited. In addition, we further investigated whether EP300 mediates histone lactylation in PTC. As confirmed by Western blot analysis, knockdown of EP300 led to a marked reduction in H3K18la levels (Figure S3D). Furthermore, we excluded any direct effects of glycolytic inhibitors such as 2-DG on EP300 expression (Figure S3E). In addition, given the report by Zhu et al. identifying KAT2A as a potential histone lactyltransferase 20, we reached the same conclusion in PTC (Figure S3F).

Rescue experiments with Nala partially restored the suppressed H3K18la levels and the reduced lactate production rate induced by 2-DG treatment (Figure 2F and Figure S4A). Consequently, Nala also partially rescued proliferation (Figure 2G; Figure 4B) and invasion (Figures 2H and 2I; Figures S4C and S4D) in PTC cells, highlighting the contribution of lactate-mediated histone lactylation to tumor malignancy.

*In vivo*, we established subcutaneous xenografts using IHH4 cells in nude mice. Once tumors reached approximately 100 mm³, mice were randomly divided into four groups (Figure 2J). Tumor growth was markedly suppressed by 2-DG treatment, and this suppression was partially reversed by Nala administration (Figures 2K and 2L; Figure S4E). Detailed IHC staining results of the xenograft tumors are presented in Figures S5A and S5B.

These findings confirm histone lactylation—particularly H3K18la—as a key regulator of PTC proliferation and invasion, underscoring its therapeutic potential.

### Lactate Accumulation Leads to Enhanced STAT1 RNA Expression by H3K18la in its Promoter Region

**Figure 3:**
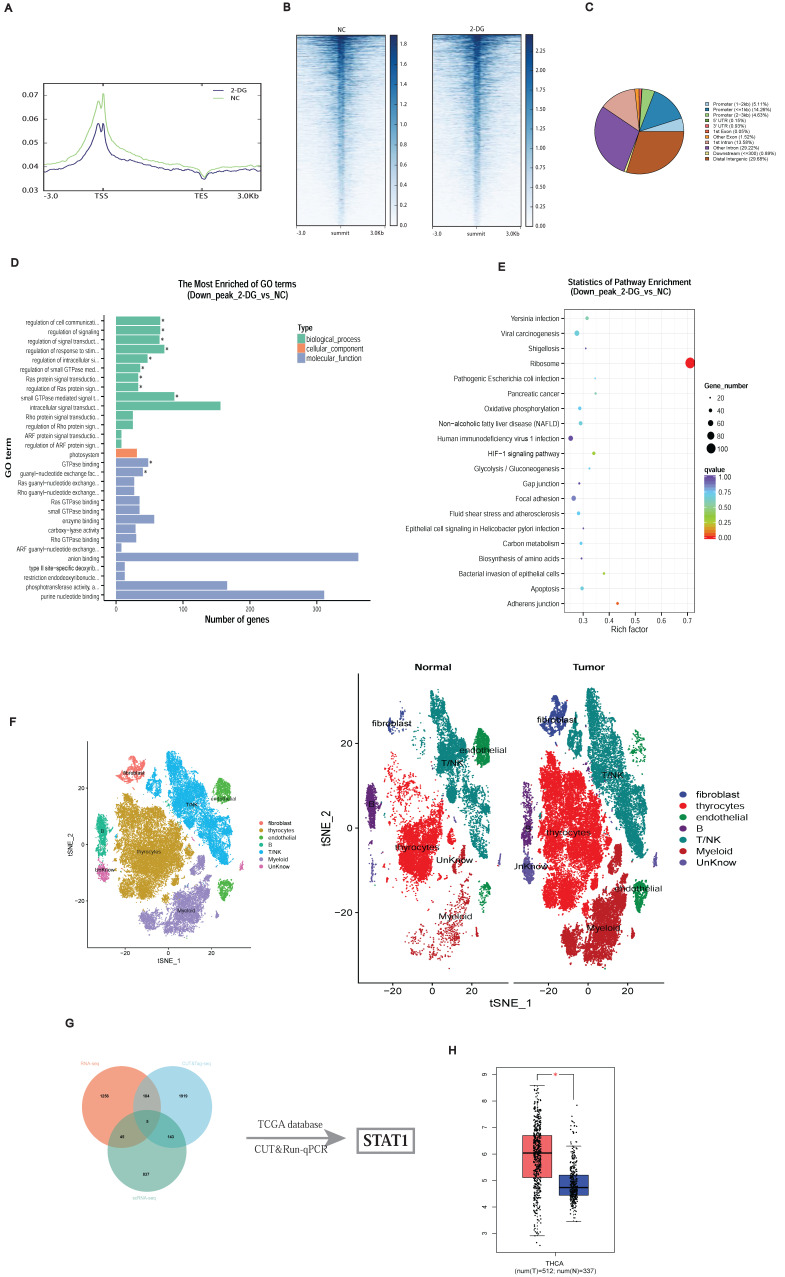
Identification of potential downstream targets of H3K18la in PTC. **(A-C)** Genomic distribution of H3K18la peaks relative to TSS. **(D-E)** GO (D) and KEGG (E) enrichment analyses of genes downregulated upon H3K18la suppression. **(F)** t-SNE plot of thyroid epithelial clusters from single-cell RNA-seq (GSE184362) of PTC and adjacent normal tissues. **(G)** Integration of CUT&Tag-seq, RNA-seq, and scRNA-seq identifies STAT1 as a candidate downstream gene. **(H)** STAT1 expression in thyroid cancer versus normal tissues from TCGA and GTEx databases.

To clarify the downstream transcriptional consequences of histone lactylation, we conducted CUT&Tag assays using an H3K18la-specific antibody, combined with subsequent CUT&Tag-seq and RNA-seq analyses. The distribution density of H3K18la-binding peaks around transcription start sites (TSSs) revealed pronounced enrichment in promoter regions (Figures 3A-3C). Kyoto Encyclopedia of Genes and Genomes (KEGG) and Gene Ontology enrichment analyses indicated that downregulated genes were predominantly involved in metabolic pathways, including Oxidative phosphorylation, Carbon metabolism, and HIF-1 signaling (Figures 3D and 3E).

Integration of single-cell RNA-seq data from three matched PTC patient samples (GSE184362) identified differentially expressed genes in thyroid epithelial cells (Figure 3F) 19. Combining CUT&Tag-seq, RNA-seq, scRNA-seq, and publicly available datasets highlighted STAT1 as a critical downstream target of H3K18la (Figure 3G; Table S1). TCGA and GTEx database analysis confirmed STAT1 upregulation in thyroid cancers (Figure 3H).

**Figure 4:**
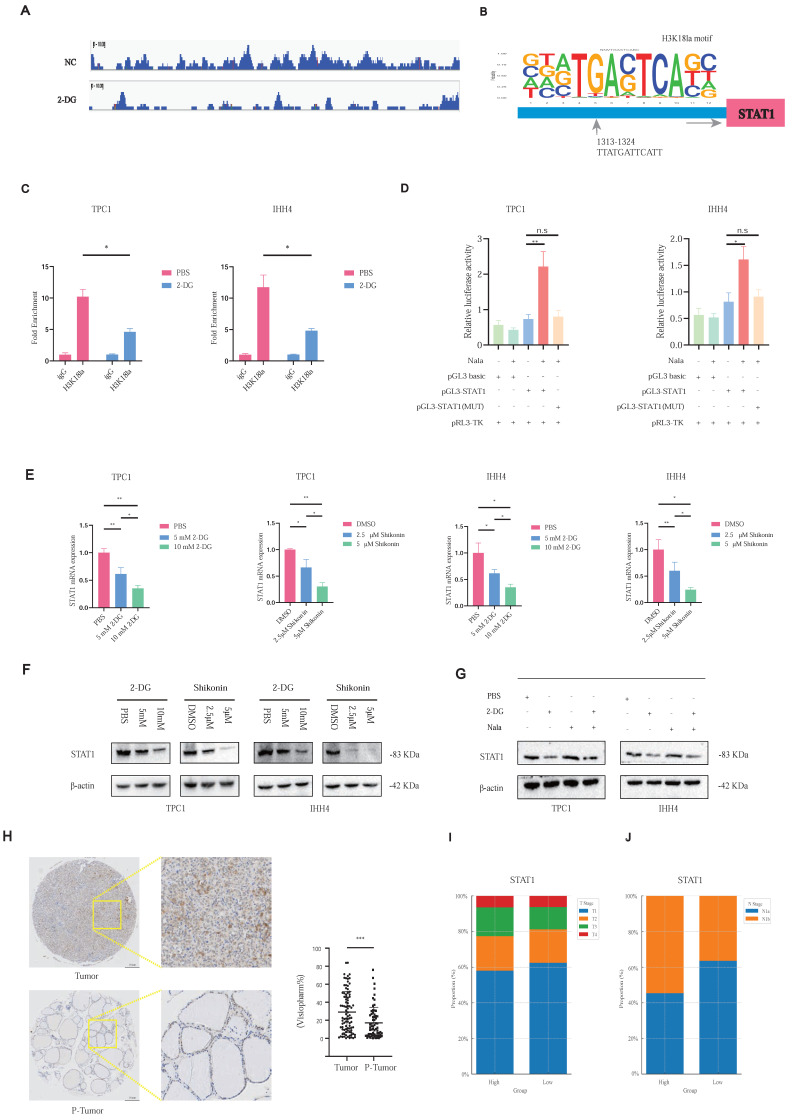
H3K18la promotes STAT1 transcription and correlates with malignant features in PTC. **(A)** Representative CUT&Tag-seq tracks showing H3K18la enrichment at the STAT1 promoter. **(B)** Conserved H3K18la-binding motif predicted from CUT&Tag-seq data. **(C)** CUT&RUN-qPCR analysis showing reduced H3K18la enrichment at the STAT1 promoter after 2-DG treatment. **(D)** Dual-luciferase reporter assay showing enhanced STAT1 promoter activity under Nala treatment. **(E-F)** mRNA (E) and protein (F) levels of STAT1 after treatment with varying concentrations of 2-DG and Shikonin. **(G)** Partial rescue of STAT1 expression by Nala following 2-DG treatment. **(H-J)** IHC staining of STAT1 in PTC TMA and its correlation with T stage (I) and N stage (J).

Notably, CUT&Tag-seq revealed pronounced H3K18la enrichment at the STAT1 promoter (Figure 4A). Motif analysis identified a conserved binding site (Figure 4B), confirmed by CUT&RUN-qPCR, showing reduced H3K18la binding upon glycolytic inhibition with 2-DG (Figure 4C). Luciferase assays revealed that STAT1 promoter activity was inducible by Nala and attenuated by 2-DG treatment, whereas this effect was abolished upon promoter mutation (Figure 4D; Figure S5C).

Both 2-DG and Shikonin treatments reduced STAT1 mRNA (Figure 4E) and protein levels (Figure 4F), with Nala partially rescuing these reductions (Figure 4G). Further, TMA-based IHC confirmed higher STAT1 expression in tumor tissues (Figure 4H), correlating with increased tumor size (Figure 4I) and cervical lymph node metastasis (Figure 4J; Figure S5D).

These findings highlight H3K18la-mediated STAT1 transcriptional activation as a central mechanism promoting PTC malignancy.

### STAT1 Functions as an Oncogenic Driver of Cell Proliferation and Invasion in PTC

**Figure 5:**
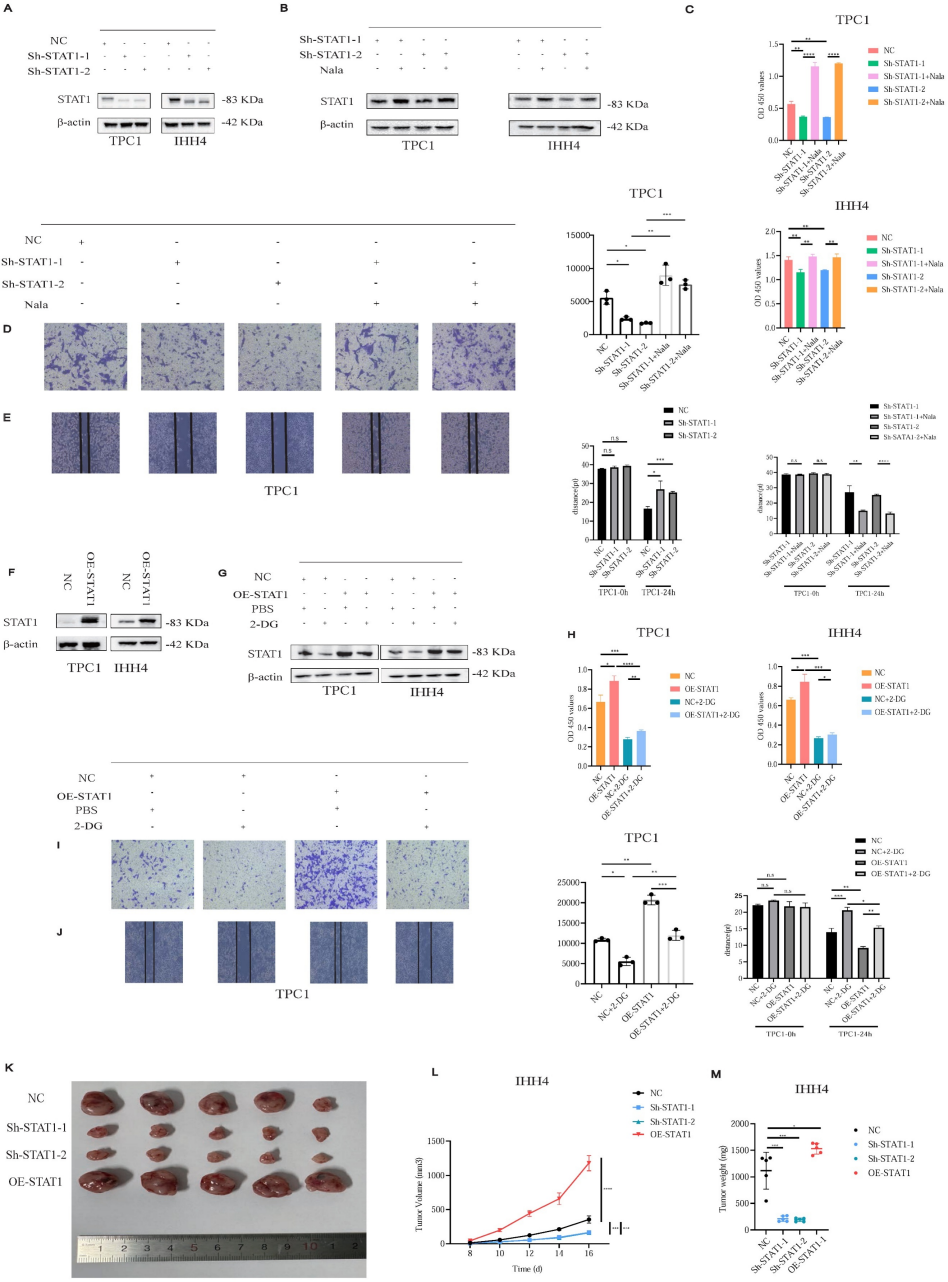
H3K18la modulates PTC cell proliferation and invasion through STAT1. **(A)** STAT1 expression in Sh-STAT1-transfected PTC cells. **(B)** Nala-induced restoration of STAT1 levels in Sh-STAT1 cells. **(C-E)** Decreased proliferation (C) and invasion (D-E) in Sh-STAT1 cells, partially reversed by Nala. **(F)** STAT1 expression in OE-STAT1-transfected PTC cells. **(G)** Suppression of STAT1 levels in OE-STAT1 cells after 2-DG treatment. **(H-J)** Inhibition of proliferation (H) and invasion (I-J) in OE-STAT1 cells by 2-DG. **(K-M)** Tumor images (K), growth curves (L), and weight comparisons (M) in xenograft models with different STAT1 expression levels.

Given STAT1's regulation by H3K18la, we established stable STAT1 knockdown (Sh-STAT1) and overexpression (OE-STAT1) PTC cell lines (Figures 5A and 5F). Nala treatment partially restored suppressed STAT1 expression in Sh-STAT1 cells (Figure 5B), correspondingly rescuing reduced proliferation and invasion (Figures 5C-5E; Figures S6A-S6C). Conversely, enhanced proliferation and invasion in OE-STAT1 cells were attenuated by 2-DG-induced glycolytic inhibition (Figures 5G-5J; Figures S7A-S7C). In addition, based on data from the TCGA and GTEx databases, as well as comparative analyses between PTC cell lines and normal thyroid epithelial cells, we explored the expression patterns of other STAT family members and found that, among the STAT family, only STAT1 exhibited a pro-tumorigenic role in PTC (Figure S8A and S8B).

In xenograft models, Sh-STAT1 tumors were significantly smaller, whereas OE-STAT1 tumors exhibited accelerated growth relative to controls (Figures 5K-5M; Figure S9A).

Collectively, STAT1 functions as an oncogenic driver in PTC, modulating malignant behaviors through lactate-dependent histone lactylation.

### STAT1 Enhances lactate Production in PTC by Transcriptionally Activating LDHA

**Figure 6:**
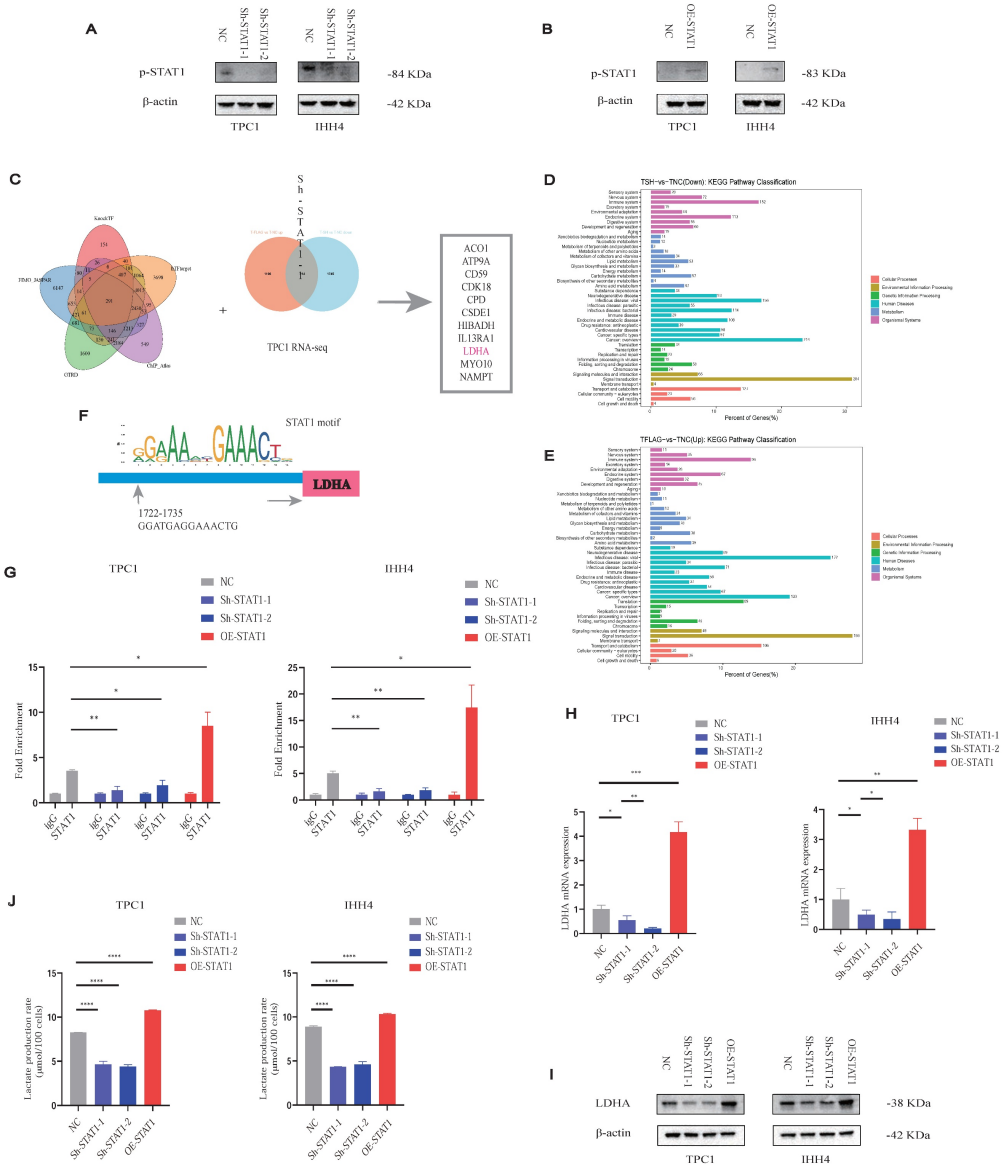
Lactate promotes PTC malignancy via the H3K18la-STAT1-LDHA axis. **(A-B)** p-STAT1 levels in Sh-STAT1 (A) and OE-STAT1 (B) cells with or without Nala or 2-DG treatment. **(C)** Predicted STAT1 target genes and differentially expressed genes from RNA-seq in TPC1 cells. **(D-E)** KEGG enrichment analysis of overlapping genes. **(F)** Predicted STAT1 motif based on the JASPAR database. **(G)** CUT&RUN-qPCR analysis of STAT1 binding at the LDHA promoter. **(H-I)** LDHA mRNA (H) and protein (I) expression in cells with altered STAT1 expression. **(J)** L-lactate production rates in PTC cells with different STAT1 expression levels.

As STAT1 typically acts via phosphorylation (p-STAT1), we verified that p-STAT1 expression correlated with STAT1 levels in Sh-STAT1 and OE-STAT1 cells, respectively (Figures 6A and 6B).

To delineate STAT1 transcriptional targets, we integrated five transcription factor databases with RNA-seq data from TPC1 Sh-STAT1 and OE-STAT1 cells (Figure 6C; Table S2). KEGG analysis revealed significant enrichment of metabolic pathways (Figures 6D and 6E). Notably, LDHA, a rate-limiting enzyme for lactate production, was identified as a direct STAT1 target.

CUT&RUN-qPCR validated STAT1 binding to the LDHA promoter, markedly reduced in Sh-STAT1 and enhanced in OE-STAT1 cells (Figures 6F and 6G). Moreover, given the increased GLUT1 and LDHB protein levels in PTC cell lines observed in Figure 1H, we conducted additional validation; however, alterations in STAT1 expression failed to regulate the transcription of either molecule (Figure S9B and S9C). STAT1 and LDHA expression correlated positively at both mRNA (Figure 6H) and protein levels (Figure 6I), further confirmed by TCGA analyses (Figure S9D). AlphaFold3 modeling suggested potential interaction sites between STAT1 and LDHA (Figure S9E).

Consistently, OE-STAT1 cells exhibited significantly increased L-lactate production, whereas Sh-STAT1 cells showed the opposite effect (Figure 6J).

**Figure 7:**
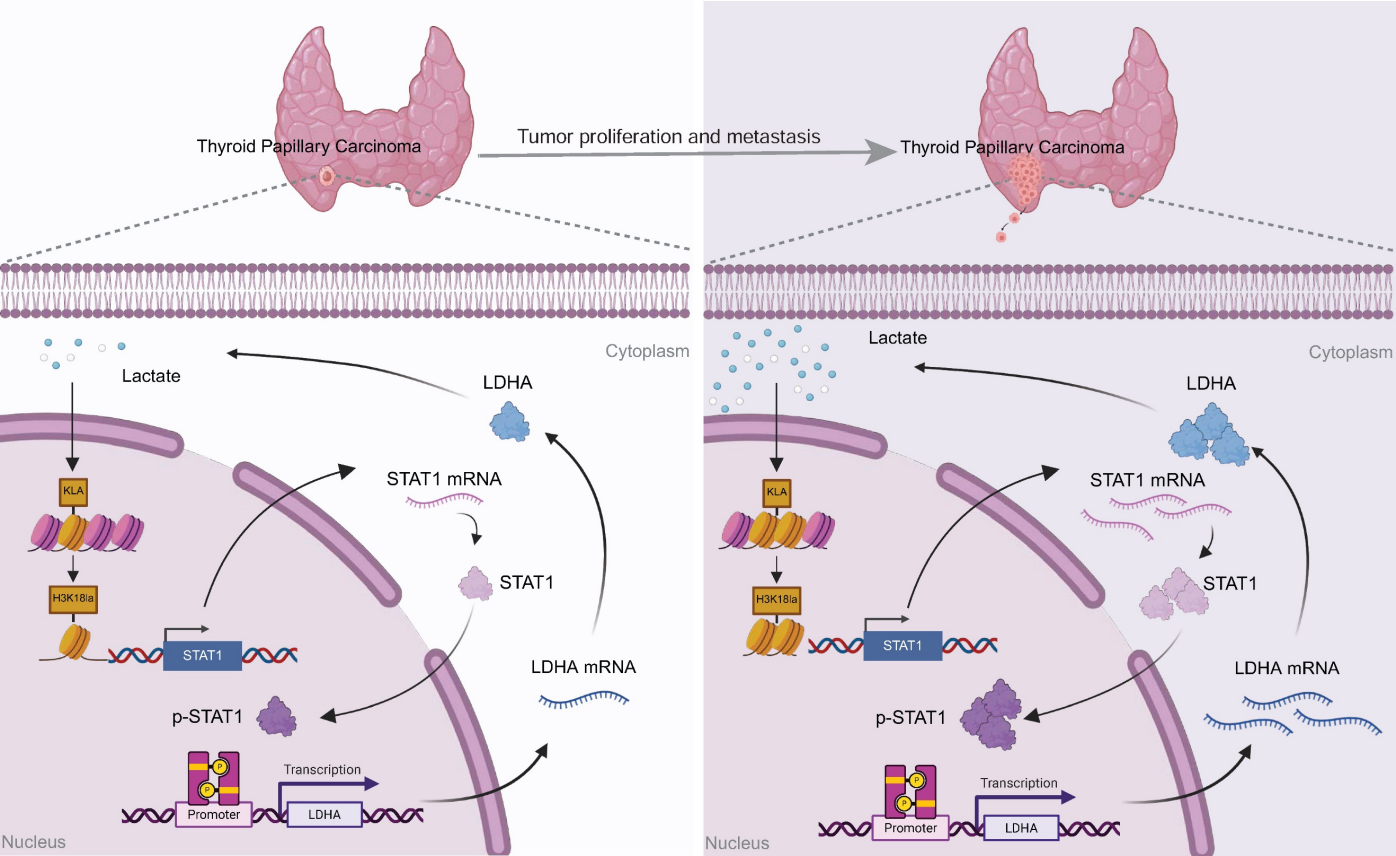
Proposed model: Tumor-derived lactate enhances H3K18la, which transcriptionally activates STAT1, thereby promoting the transcription of LDHA, increasing its expression and lactate production. This forms a self-reinforcing H3K18la-STAT1-LDHA feedback loop that drives the malignant progression of PTC.

Our findings collectively demonstrate a positive feedback mechanism wherein tumor-derived lactate promotes H3K18la-mediated STAT1 transcription, which enhances LDHA expression and lactate production. This H3K18la-STAT1-LDHA axis plays a crucial role in driving proliferation and metastasis in PTC, representing a novel therapeutic target (Figure 7).

## Discussion

Over the past decades, metabolic reprogramming has been firmly established as a hallmark of cancer, enabling tumor cells to sustain rapid proliferation, invasion, and other malignant phenotypes. Lactate, traditionally viewed as a mere metabolic byproduct of the “Warburg effect” 21, was long considered inert in terms of biological functionality. However, this paradigm fundamentally shifted following the discovery by Zhao et al. that lactate serves as a substrate for histone lactylation, a novel epigenetic modification 11.

Among the various lactylation marks identified, H3K18la has garnered significant attention due to its critical involvement in tumor progression, therapeutic resistance, and immune modulation across multiple cancer types 12-14. Despite these advances, the precise functional role and underlying mechanisms of histone lactylation in human thyroid carcinoma remain largely unexplored.

Although PTC generally exhibits favorable prognosis and high overall survival rates 22, a clinically significant subset of patients manifests aggressive behavior characterized by rapid disease progression and frequent recurrence. This clinical reality underscores an urgent need to elucidate the molecular aberrations and mechanisms driving PTC malignancy to inform the development of precise and effective therapeutic strategies. Herein, we demonstrate for the first time that tumor-derived lactate promotes PTC tumorigenesis and progression via H3K18la-mediated epigenetic remodeling, thereby establishing a direct mechanistic link between altered tumor metabolism and epigenetic dysregulation. This finding not only advances our mechanistic understanding of thyroid cancer biology but also provides crucial evidence supporting the emerging concept of metabolic-epigenetic interplay. Furthermore, identification of this lactylation-dependent regulatory axis presents potential therapeutic avenues for targeting aggressive and treatment-refractory forms of PTC, underscoring its translational significance.

To investigate the molecular mechanisms by which H3K18la exerts its tumor-promoting effects in PTC, we employed CUT&Tag, an advanced chromatin profiling technology offering higher resolution, enhanced signal-to-noise ratio, and reduced background compared to conventional chromatin immunoprecipitation sequencing (ChIP-seq) 23. Integration of CUT&Tag data identified STAT1 as a candidate downstream target gene regulated by H3K18la in PTC.

STAT1, widely recognized as a critical signal transducer and transcription factor, plays complex and context-dependent roles in cancer biology and immune regulation. Classically viewed as a tumor suppressor, activated STAT1 can induce expression of anti-tumor genes and enhance the cytotoxic functions of natural killer NK cells and T cells, thereby suppressing tumor growth and promoting apoptosis 24-26. However, accumulating evidence also associates elevated STAT1 and phosphorylated STAT1 (p-STAT1) levels with unfavorable clinical outcomes in diverse cancer types 27, 28, potentially due to STAT1-driven expression of proinflammatory cytokines and chemokines 29. A recent study by Ge et al. highlighted that increased STAT1 expression in anaplastic thyroid carcinoma (ATC) is positively correlated with enhanced immune infiltration 30. Nevertheless, research on STAT1 in PTC remains predominantly computational, and its precise biological functions and regulatory mechanisms have not yet been experimentally defined.

To fill this knowledge gap, we conducted a comprehensive experimental analysis using a custom-constructed TMA. Our findings reveal that STAT1 is transcriptionally regulated by H3K18la and exerts a pro-tumorigenic function in PTC. Elevated STAT1 expression in tumor tissues correlated positively with adverse clinicopathological features, supporting its role as a potential oncogenic factor in thyroid cancer.

Activation of STAT1 via phosphorylation at Tyr701 is well-characterized in cancer biology, facilitating its nuclear translocation and subsequent transcriptional activity 31. By combining transcription factor prediction analyses with our RNA sequencing datasets from PTC cells, we unexpectedly identified LDHA as a STAT1-regulated target gene. Previous studies in prostate cancer demonstrated that STAT1 could enhance tumor progression through transcriptional regulation of LDHA and LDHB, highlighting its significant role in metabolic regulation 32, underscoring its role in metabolic regulation. LDHA catalyzes the critical terminal reaction of aerobic glycolysis, converting pyruvate into lactate 33, and is frequently employed as a surrogate marker for modulating cellular lactylation levels 13, 14.

In the present study, we further validate that STAT1 directly binds to and activates the LDHA promoter, significantly enhancing LDHA expression and thus increasing lactate production. This creates a positive feedback loop wherein tumor-derived lactate promotes H3K18la modification, which subsequently elevates STAT1 and LDHA expression. This self-reinforcing lactate-H3K18la-STAT1-LDHA axis not only enhances lactate accumulation but also sustains metabolic reprogramming and malignant progression in PTC.

Collectively, our findings indicate that histone lactylation and specifically H3K18la are significantly upregulated in clinical PTC samples, with levels correlating positively with aggressive tumor features. Mechanistically, we demonstrate that H3K18la directly promotes STAT1 transcription, which subsequently induces LDHA expression, enhancing lactate synthesis. Thus, tumor-derived lactate continuously fuels this epigenetic-metabolic feedback loop, driving sustained tumor growth and progression.

Our research group has long focused on elucidating the role of histone lactylation in thyroid carcinoma. Previously, we reported that BRAFV600E-driven aerobic glycolysis reshapes the lactylation landscape, contributing to enhanced proliferation in ATC 34. Future investigations will explore additional histone modifications and their phenotypic implications, further expanding our understanding of the complex epigenetic mechanisms governing thyroid cancer pathogenesis.

## Conclusion

In conclusion, this study comprehensively delineates the molecular mechanisms underlying lactate-driven malignant progression in PTC via epigenetic modulation. We demonstrate that tumor-derived lactate facilitates the establishment of a self-sustaining positive feedback loop through activation of the H3K18la-STAT1-LDHA axis, thereby continuously amplifying lactate production and promoting tumor aggressiveness. These findings provide novel mechanistic insights into the metabolic-epigenetic interplay in cancer biology and offer a robust theoretical basis for the development of therapeutic strategies that integrate metabolic reprogramming and epigenetic regulation. Specifically, pharmacologic targeting of H3K18la modification and the STAT1 signaling pathway emerges as a promising therapeutic approach for patients exhibiting aggressive and refractory PTC phenotypes, with substantial potential for clinical translation.

## Supplementary Material

Supplementary figures 1-9.

Supplementary table 1.

Supplementary table 2.

## Figures and Tables

**Table 1:** Primer sequences

Name	Sequence (5ʹ-3ʹ)
β-actin forward	GATCATTGCTCCTCCTGAGC
β-actin reverse	ACTCCTGCTTGCTGATCCAC
STAT1 forward	CAGCTTGACTCAAAATTCCTGGA
STAT1 reverse	TGAAGATTACGCTTGCTTTTCCT
LDHA forward	ATGGCAACTCTAAAGGATCAGC
LDHA reverse	CCAACCCCAACAACTGTAATCT
EP300 forward	AGCCAAGCGGCCTAAACTC
EP300 reverse	TCACCACCATTGGTTAGTCCC
H3K18la CUT&Run-qPCR forward	TATGTGACTGGCTTCCTT
H3K18la CUT&Run-qPCR reverse	TTGTTTACCAATGTTTCC
STAT1 CUT&Run-qPCR forward	ACAGGGATGAAGAAGAAACA
STAT1 CUT&Run-qPCR reverse	GCTGGGAGTCTAAGTAAGGT
GLUT1 CUT&Run-qPCR forward	TGCTTTTGTAACTACAGGC
GLUT1 CUT&Run-qPCR reverse	ATGGTAATACATATAGGTATTTCCA
LDHB CUT&Run-qPCR forward	AAGAACCCCTAAAATGGA
LDHB CUT&Run-qPCR reverse	ATGTTATGCTATCTCCAA
siRNA NC forward	UUCUCCGAACGUGUCACGU TT
siRNA NC reverse	ACGUGACACGUUCGGAGAA TT
Hsa-EP300-si-1 forward	GAUGAAUUAAUCAACUCUATT
Hsa-EP300-si-1 reverse	UAGAGUUGAUUAAUUCAUCTT
Hsa-KAT2A-si-1 forward	GCGCAUGCCUAAGGAGUAUAUTT
Hsa-KAT2A-si-1 reverse	AUAUACUCCUUAGGCAUGCGCTT
Sh-STAT1-1	GAGCAGGTTCACCAGCTTTAT
Sh-STAT1-2	TGCCAGCCTGGTTTGGTAATT
OE-STAT1	TGTCTCAGTGGTACGAACTTCAGCAGCTTGACTCAAAATTCCTGGAGCAGGTTCACCAGCTTTATGATGACAGTTTTCCCATGGAAATCAGACAGTACCTGGCACAGTGGTTAGAAAAGCAAGACTGGGAGCACGCTGCCAATGATGTTTCATTTGCCACCATCCGTTTTCATGACCTCCTGTCACAGCTGGATGATCAATATAGTCGCTTTTCTTTGGAGAATAACTTCTTGCTACAGCATAACATAAGGAAAAGCAAGCGTAATCTTCAGGATAATTTTCAGGAAGACCCAATCCAGATGTCTATGATCATTTACAGCTGTCTGAAGGAAGAAAGGAAAATTCTGGAAAACGCCCAGAGATTTAATCAGGCTCAGTCGGGGAATATTCAGAGCACAGTGATGTTAGACAAACAGAAAGAGCTTGACAGTAAAGTCAGAAATGTGAAGGACAAGGTTATGTGTATAGAGCATGAAATCAAGAGCCTGGAAGATTTACAAGATGAATATGACTTCAAATGCAAAACCTTGCAGAACAGAGAACACGAGACCAATGGTGTGGCAAAGAGTGATCAGAAACAAGAACAGCTGTTACTCAAGAAGATGTATTTAATGCTTGACAATAAGAGAAAGGAAGTAGTTCACAAAATAATAGAGTTGCTGAATGTCACTGAACTTACCCAGAATGCCCTGATTAATGATGAACTAGTGGAGTGGAAGCGGAGACAGCAGAGCGCCTGTATTGGGGGGCCGCCCAATGCTTGCTTGGATCAGCTGCAGAACTGGTTCACTATAGTTGCGGAGAGTCTGCAGCAAGTTCGGCAGCAGCTTAAAAAGTTGGAGGAATTGGAACAGAAATACACCTACGAACATGACCCTATCACAAAAAACAAACAAGTGTTATGGGACCGCACCTTCAGTCTTTTCCAGCAGCTCATTCAGAGCTCGTTTGTGGTGGAAAGACAGCCCTGCATGCCAACGCACCCTCAGAGGCCGCTGGTCTTGAAGACAGGGGTCCAGTTCACTGTGAAGTTGAGAGATGTGAATGAGAGAAATACAGTAAAAGGATTTAGGAAGTTCAACATTTTGGGCACGCACACAAAAGTGATGAACATGGAGGAGTCCACCAATGGCAGTCTGGCGGCTGAATTTCGGCACCTGCAATTGAAAGAACAGAAAAATGCTGGCACCAGAACGAATGAGGGTCCTCTCATCGTTACTGAAGAGCTTCACTCCCTTAGTTTTGAAACCCAATTGTGCCAGCCTGGTTTGGTAATTGACCTCGAGACGACCTCTCTGCCCGTTGTGGTGATCTCCAACGTCAGCCAGCTCCCGAGCGGTTGGGCCTCCATCCTTTGGTACAACATGCTGGTGGCGGAACCCAGGAATCTGTCCTTCTTCCTGACTCCACCATGTGCACGATGGGCTCAGCTTTCAGAAGTGCTGAGTTGGCAGTTTTCTTCTGTCACCAAAAGAGGTCTCAATGTGGACCAGCTGAACATGTTGGGAGAGAAGCTTCTTGGTCCTAACGCCAGCCCCGATGGTCTCATTCCGTGGACGAGGTTTTGTAAGGAAAATATAAATGATAAAAATTTTCCCTTCTGGCTTTGGATTGAAAGCATCCTAGAACTCATTAAAAAACACCTGCTCCCTCTCTGGAATGATGGGTGCATCATGGGCTTCATCAGCAAGGAGCGAGAGCGTGCCCTGTTGAAGGACCAGCAGCCGGGGACCTTCCTGCTGCGGTTCAGTGAGAGCTCCCGGGAAGGGGCCATCACATTCACATGGGTGGAGCGGTCCCAGAACGGAGGCGAACCTGACTTCCATGCGGTTGAACCCTACACGAAGAAAGAACTTTCTGCTGTTACTTTCCCTGACATCATTCGCAATTACAAAGTCATGGCTGCTGAGAATATTCCTGAGAATCCCCTGAAGTATCTGTATCCAAATATTGACAAAGACCATGCCTTTGGAAAGTATTACTCCAGGCCAAAGGAAGCACCAGAGCCAATGGAACTTGATGGCCCTAAAGGAACTGGATATATCAAGACTGAGTTGATTTCTGTGTCTGAAGTTCACCCTTCTAGACTTCAGACCACAGACAACCTGCTCCCCATGTCTCCTGAGGAGTTTGACGAGGTGTCTCGGATAGTGGGCTCTGTAGAATTCGACAGTATGATGAACACAGTATAG

**Table 2:** Antibodies

Name	Article numbers (brand)
HKII	ab104836(abcam)
PKM2	ab137852(abcam)
GLUT1	ab14683(abcam)
LDHA	DF6280(Affinity Biosciences)
LDHB	DF12101(Affinity Biosciences)
H3K9la	PTM-1419RM(PTM Biosciences)
H3K14la	PTM-1414RM(PTM Biosciences)
H3K18la	PTM-1406RM(PTM Biosciences)
H3K27la	PTM-1428(PTM Biosciences)
H4K12la	PTM-1411RM(PTM Biosciences)
Lys-La	PTM-1401RM(PTM Biosciences)
STAT1	A12075(abclonal)
STAT2	A3588 (abclonal)
STAT3	A19566(abclonal)
STAT4	A4523(abclonal)
STAT5A	A21228(abclonal)
STAT5B	A19567(abclonal)
STAT6	A19120(abclonal)
p-STAT1	#7649(CST)
β-actin	81,115-1-RR (Proteintech)
KAT2A	66575-1-Ig (Proteintech)
EP300	#86377(CST)
Histone H3	sc-517576(Santa Cruz)
secondary antibodies	AS014 (Abclonal), SA00001-1 (Proteintech)
