# Supplementary material for: Tumor-Derived Lactate Drives Malignant Progression of Refractory Papillary Thyroid Carcinoma via the H3K18la-STAT1-LDHA Axis
Source: Int J Biol Sci. 2025 Oct 1;21(14):6373–88. doi: 10.7150/ijbs.120277 (PMC12594596; doi:10.7150/ijbs.120277)
Supplement: Supplementary file 1 — Supplementary figures 1-9. [file ijbsv21p6373s1.pdf]

Supplementary Figure legends:

Figure S1:

S1A: IHC results of pan-lysine lactylation based on TMA.

S1B: IHC results of H3K18la based on TMA.

S1C: Correlation between pan-lysine lactylation and H3K18la IHC scores for each sample based on TMA.

S1D: IHC results of H3K9la based on TMA.

S1E: Correlation between pan-lysine lactylation and H3K9la IHC scores for each sample based on TMA.

S1F: IHC results of H4K12la based on TMA.

S1G: Correlation between pan-lysine lactylation and H4K12la IHC scores for each sample based on TMA.

S1H and S1I: H4K12la IHC staining in PTC samples stratified by T stage (S1H) and N stage (S1I).

Figure S2:

S2A: IHC results of H3K18ac based on TMA.

S2B and S2C: H3K18ac IHC staining in PTC samples stratified by T stage (S2B) and N stage (S2C).

S2D: IHC results of H3K18cr based on TMA.

S2E and S2F: H3K18cr IHC staining in PTC samples stratified by T stage (S2E) and N stage (S2F).

Figure S3:

S3A: Proliferation levels of PTC cell lines following treatment with 2-DG and Shikonin.

S3B and S3C: Invasion levels of PTC cell lines following treatment with 2-DG and Shikonin.

S3D: Levels of H3K18la following EP300 knockdown.

S3E: mRNA and protein expression levels of EP300 in PTC cell lines after treatment with 2-DG and Shikonin.

S3F: Levels of H3K18la following KAT2A knockdown.

Figure S4:

S4A: Lactate production rate in PTC cell lines following treatment with 2-DG and Nala.

S4B: Proliferation levels of PTC cell lines following treatment with 2-DG and Nala.

S4C and S4D: Invasion levels of PTC cell lines following treatment with 2-DG and Nala.

S4E: Tumor images of subcutaneous xenografts in PTC cells following treatment with 2-DG and Nala.

Figure S5:

S5A: IHC results of H3K18la in subcutaneous PTC xenografts under 2-DG and Nala treatment;

S5B: IHC results of STAT1 in subcutaneous PTC xenografts under 2-DG and Nala treatment;

S5C: Dual-luciferase reporter assay showing suppressed STAT1 promoter activity under 2-DG treatment;

S5D: IHC results of STAT1 based on TMA.

Figure S6:

S6A: Proliferation levels of PTC cell lines with Sh-STAT1 and after Nala treatment;
S6B and S6C: Invasion levels of PTC cell lines with Sh-STAT1 and after Nala treatment.

Figure S7:
S7A: Proliferation levels of PTC cell lines with OE-STAT1 and after 2-DG treatment;
S6B and S6C: Invasion levels of PTC cell lines with OE-STAT1 and after 2-DG treatment.

Figure S8:
S8A: Expression levels of STAT family members other than STAT1 in PTC tumor tissues compared to p-tumor tissues based on the TCGA database.
S8B: Protein expression levels of STAT family members other than STAT1 in PTC cell lines compared with normal thyroid epithelial cells.

Figure S9:
S9A: IHC results of STAT1 in subcutaneous xenograft tumors derived from PTC cells with Sh-STAT1 or OE-STAT1.
S9B: CUT&RUN-qPCR results of STAT1 binding at the GLUT1 promoter.
S9C: CUT&RUN-qPCR results of STAT1 binding at the LDHB promoter.
S9D: Gene co-expression analysis of STAT1 and LDHA based on the TCGA database.
S9E: Predicted potential interaction sites between STAT1 and LDHA based on AlphaFold3.

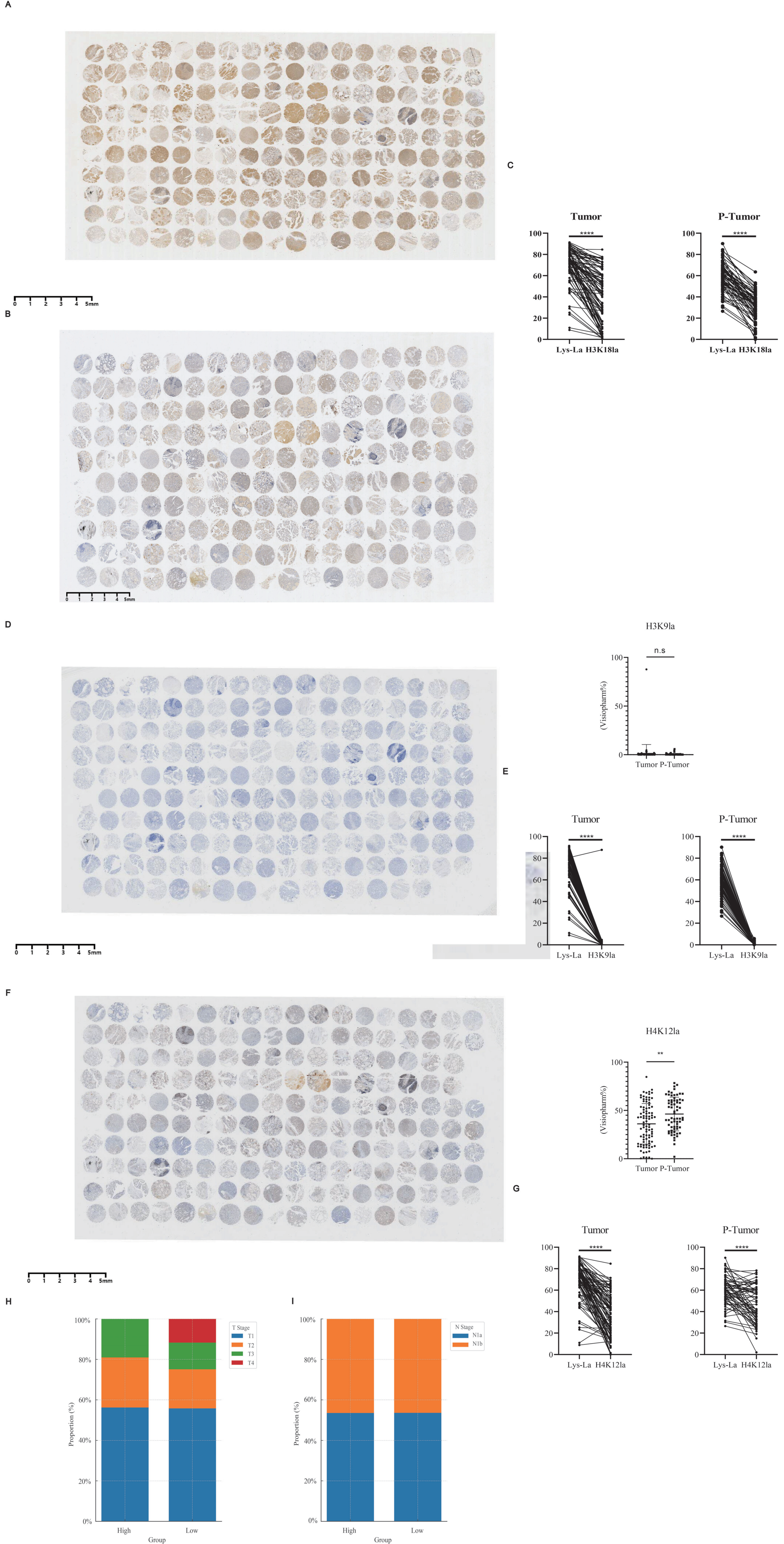

A
B
C
Tumor
P-Tumor
****
100
80
60
40
20
0
Lys-La H3K18la
D
H3K9la
n.s
(Visiopharm%)
Tumor P-Tumor
E
Lys-La H3K9la
F
H4K12la
**
G
Lys-La H4K12la
0 1 2 3 4 5mm
H
T Stage
T1
T2
T3
T4
Proportion (%)
High
Low
Group
I
N Stage
N1a
N1b

A

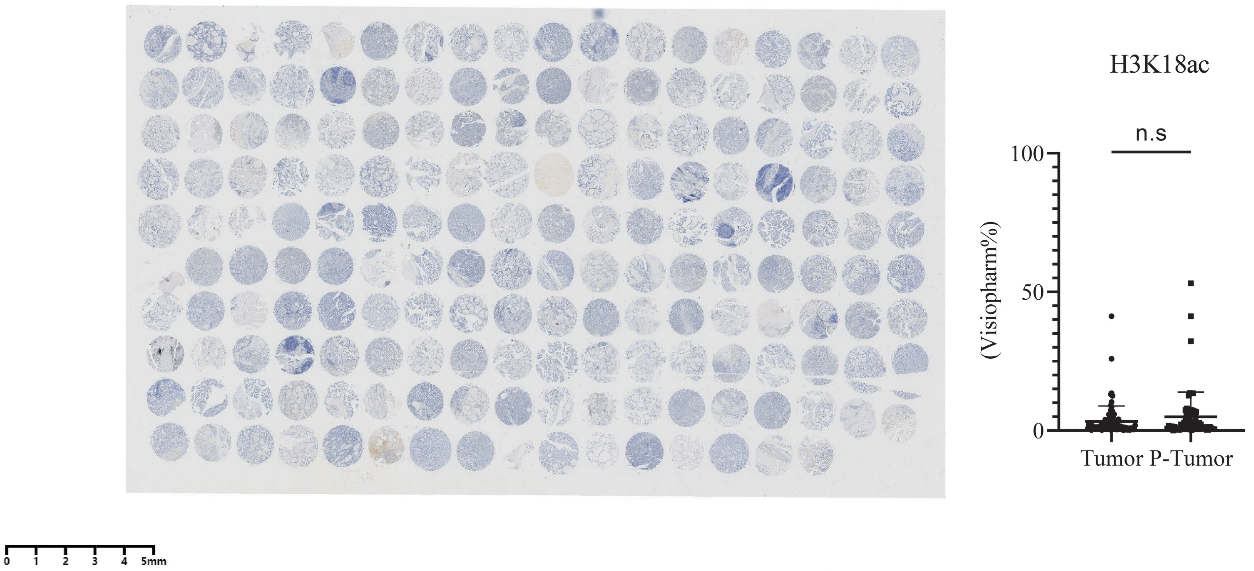
H3K18ac
n.s
100
50
0
(Visiopharm%)
Tumor P-Tumor
0 1 2 3 4 5mm

B`

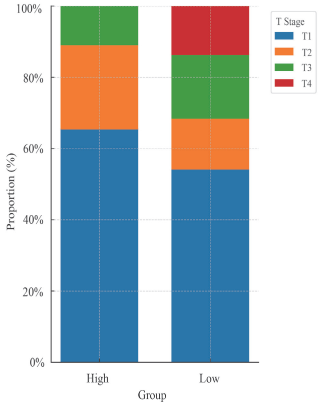
100%
80%
60%
40%
20%
0%
Proportion (%)
T Stage
T1
T2
T3
T4
High
Low
Group

C

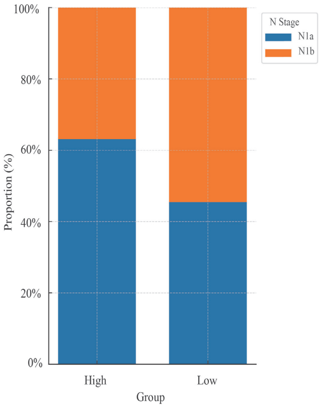
100%
80%
60%
40%
20%
0%
Proportion (%)
N Stage
N1a
N1b
High
Low
Group

D

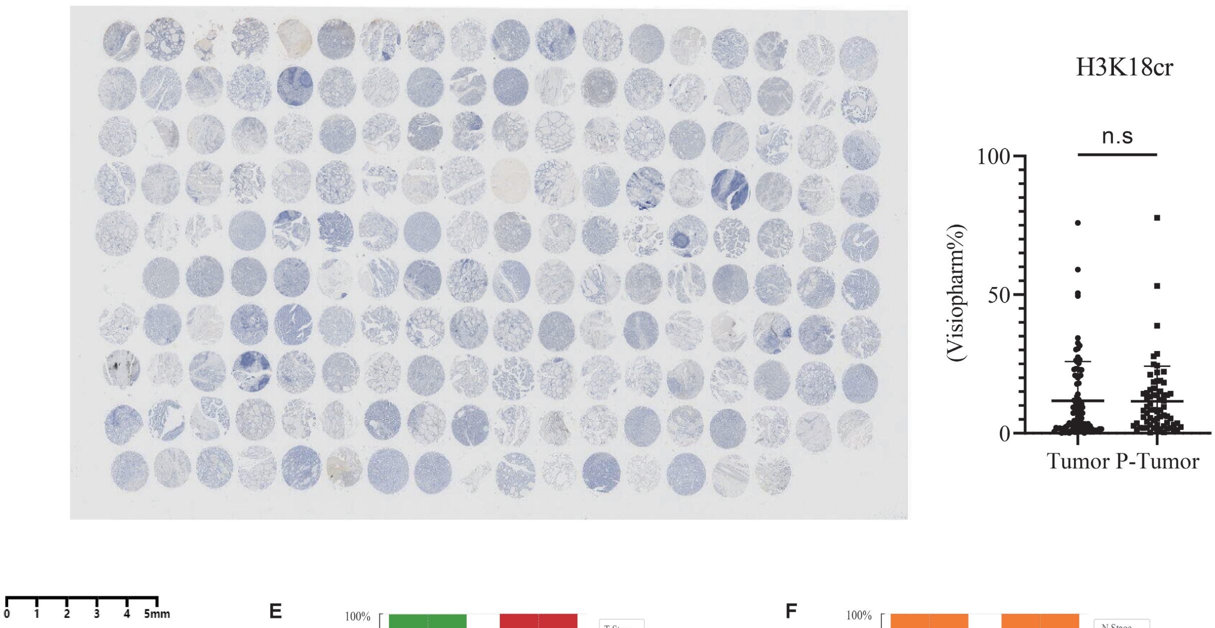
H3K18cr
n.s
100
50
0
(Visiopharm%)
Tumor P-Tumor
0 1 2 3 4 5mm

E

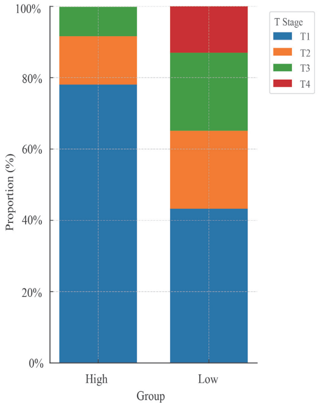
100%
80%
60%
40%
20%
0%
Proportion (%)
T Stage
T1
T2
T3
T4
High
Low
Group

F

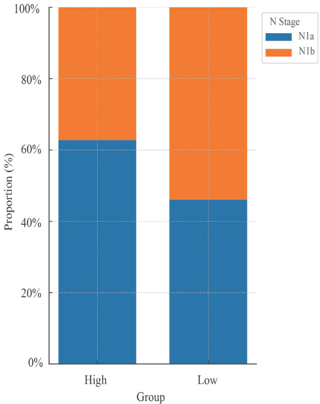
100%
80%
60%
40%
20%
0%
Proportion (%)
N Stage
N1a
N1b
High
Low
Group

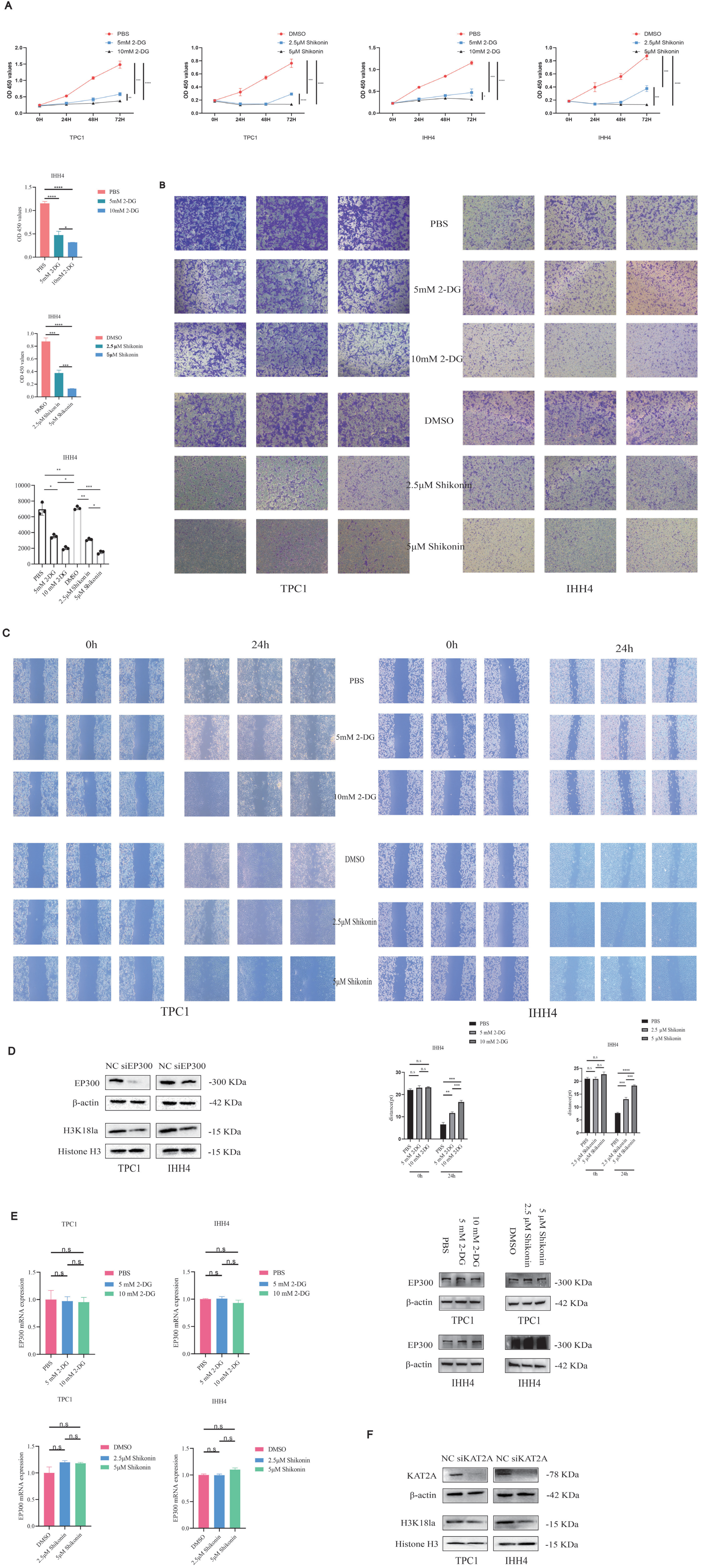
A
PBS
5mM 2-DG
10mM 2-DG
DMSO
2.5μM Shikonin
5μM Shikonin
OD 450 values
0H
24H
48H
72H
TPC1
IHH4
B
PBS
5mM 2-DG
10mM 2-DG
DMSO
2.5μM Shikonin
5μM Shikonin
TPC1
IHH4
C
0h
24h
PBS
5mM 2-DG
10mM 2-DG
DMSO
2.5μM Shikonin
5μM Shikonin
TPC1
IHH4
D
NC siEP300 NC siEP300
EP300
β-actin
H3K18la
Histone H3
-300 KDa
-42 KDa
-15 KDa
-15 KDa
TPC1
IHH4
distance(px)
E
EP300 mRNA expression
n.s
PBS
5 mM 2-DG
10 mM 2-DG
DMSO
2.5μM Shikonin
5μM Shikonin
TPC1
IHH4
EP300
β-actin
-300 KDa
-42 KDa
F
NC siKAT2A NC siKAT2A
KAT2A
β-actin
H3K18la
Histone H3
-78 KDa
-42 KDa
-15 KDa
-15 KDa
TPC1
IHH4

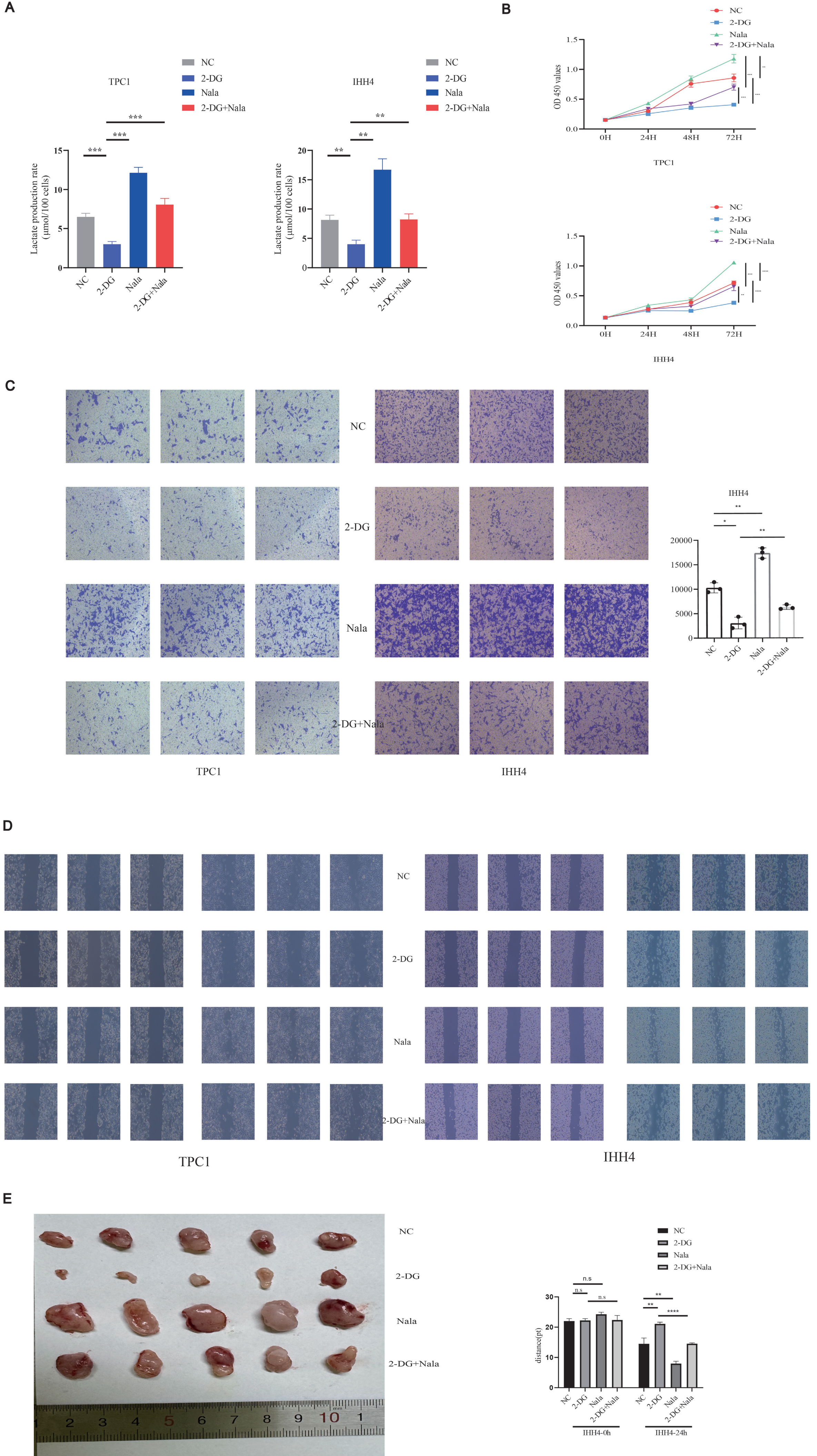
A
TPC1
IHH4
NC
2-DG
Nala
2-DG+Nala
Lactate production rate (μmol/100 cells)
B
OD 450 values
0H
24H
48H
72H
C
D
TPC1
IHH4
E
distance(pt)
IHH4-0h
IHH4-24h
n.s

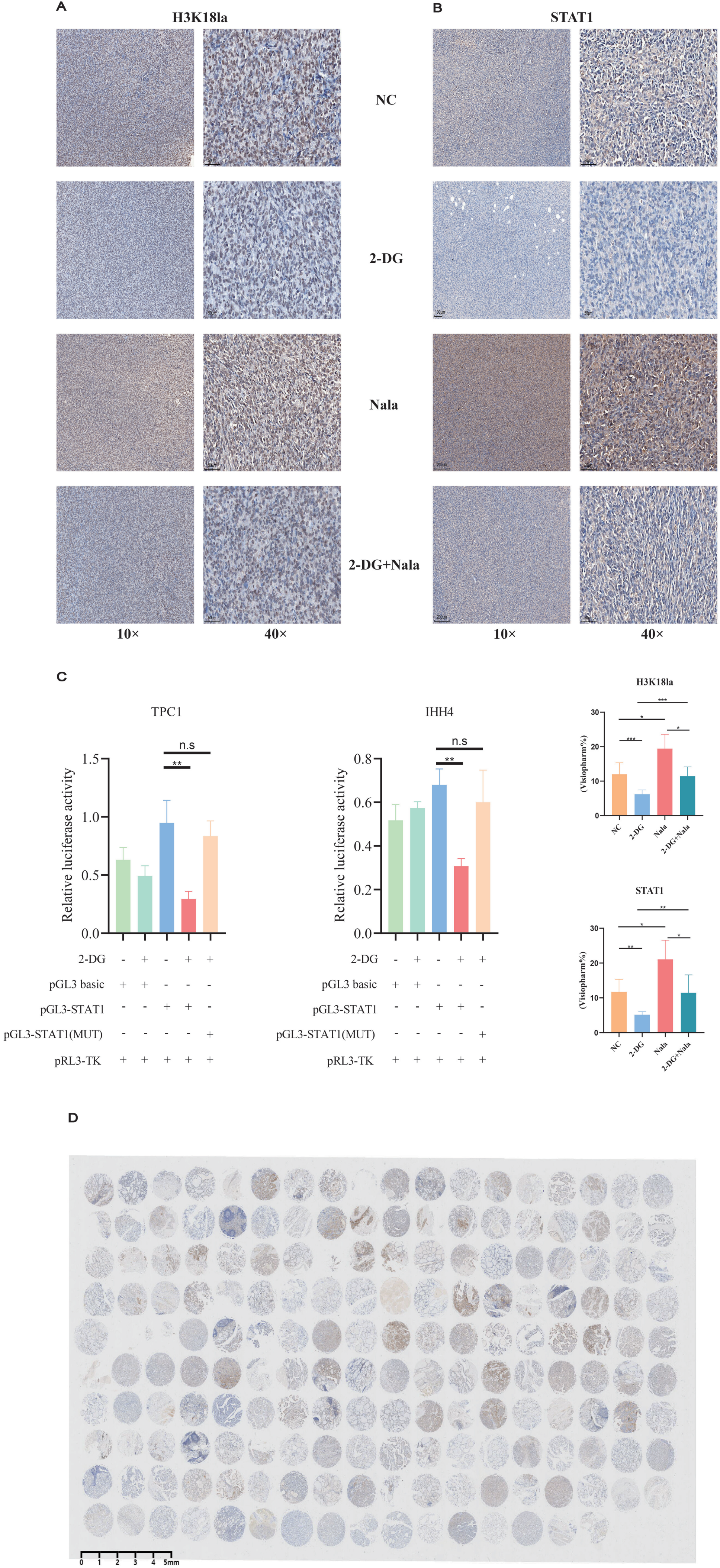
A
H3K18la
B
STAT1
NC
2-DG
Nala
2-DG+Nala
10×
40×
10×
40×
C
TPC1
IHH4
Relative luciferase activity
n.s
**
2-DG
pGL3 basic
pGL3-STAT1
pGL3-STAT1(MUT)
pRL3-TK
H3K18la
STAT1
(Visiopharm%)
NC
2-DG
Nala
2-DG+Nala
D
0 1 2 3 4 5mm

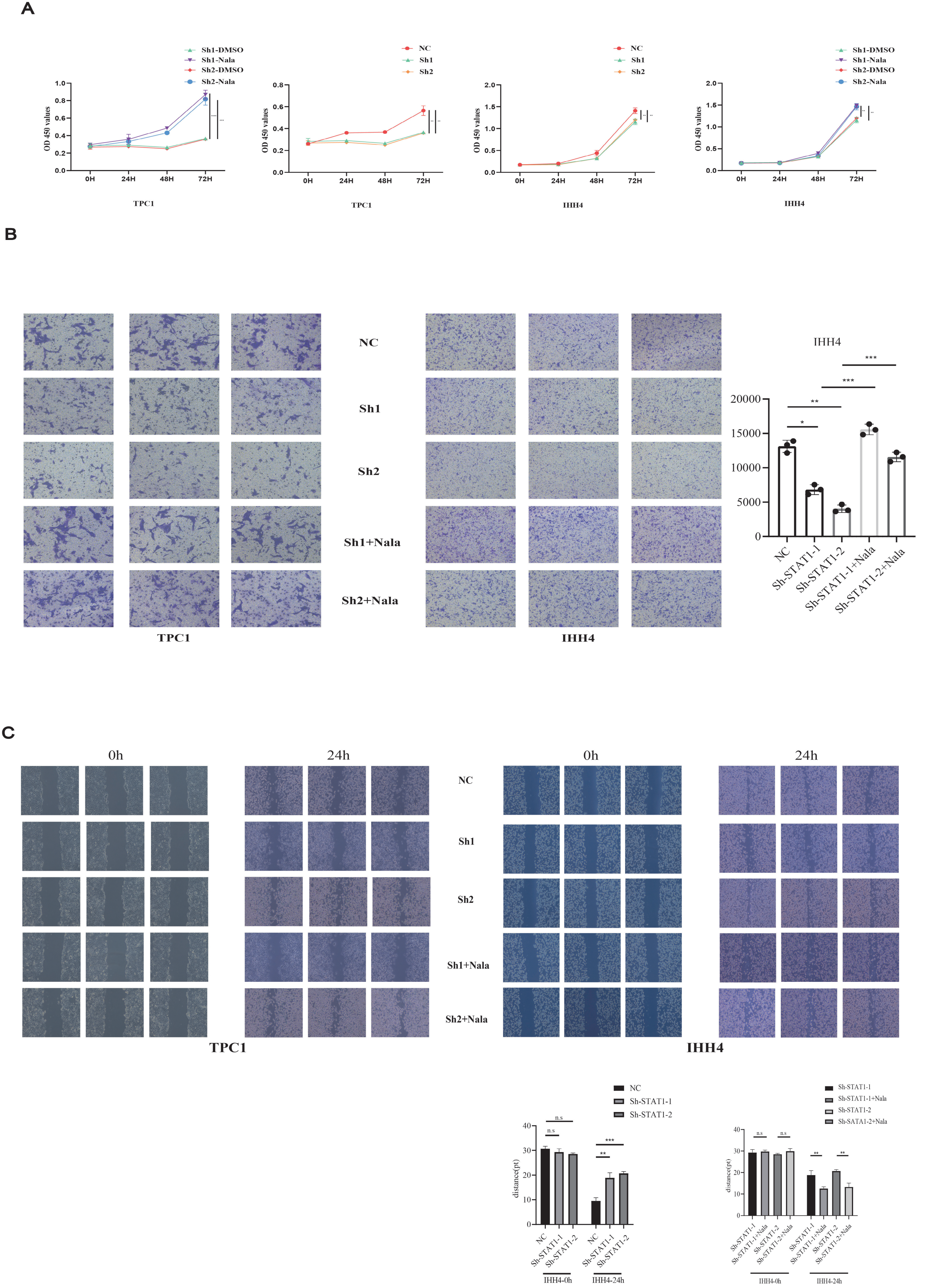
A
Sh1-DMSO
Sh1-Nala
Sh2-DMSO
Sh2-Nala
NC
Sh1
Sh2
OD 450 values
0H
24H
48H
72H
TPC1
IHH4
B
NC
Sh1
Sh2
Sh1+Nala
Sh2+Nala
TPC1
IHH4
NC
Sh-STAT1-1
Sh-STAT1-2
Sh-STAT1-1+Nala
Sh-STAT1-2+Nala
C
0h
24h
NC
Sh1
Sh2
Sh1+Nala
Sh2+Nala
TPC1
IHH4
distance(pt)
n.s
IHH4-0h
IHH4-24h
Sh-STAT1-1+Nala
Sh-SATA1-2+Nala

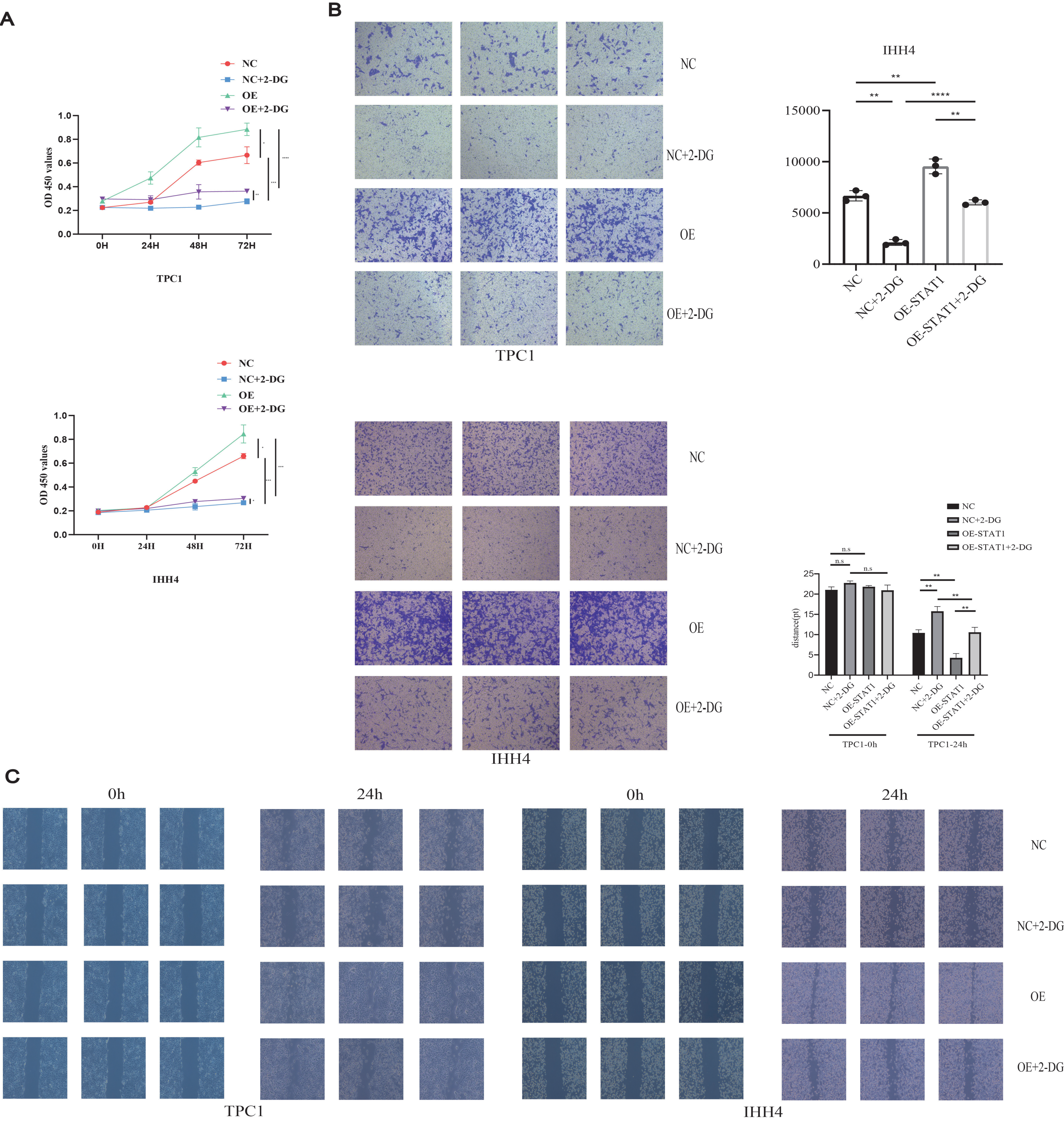

A
NC
NC+2-DG
OE
OE+2-DG
OD 450 values
0H
24H
48H
72H
TPC1
IHH4
B
NC
NC+2-DG
OE
OE+2-DG
TPC1
IHH4
OE-STAT1
OE-STAT1+2-DG
distance(pt)
TPC1-0h
TPC1-24h
n.s
C
0h
24h

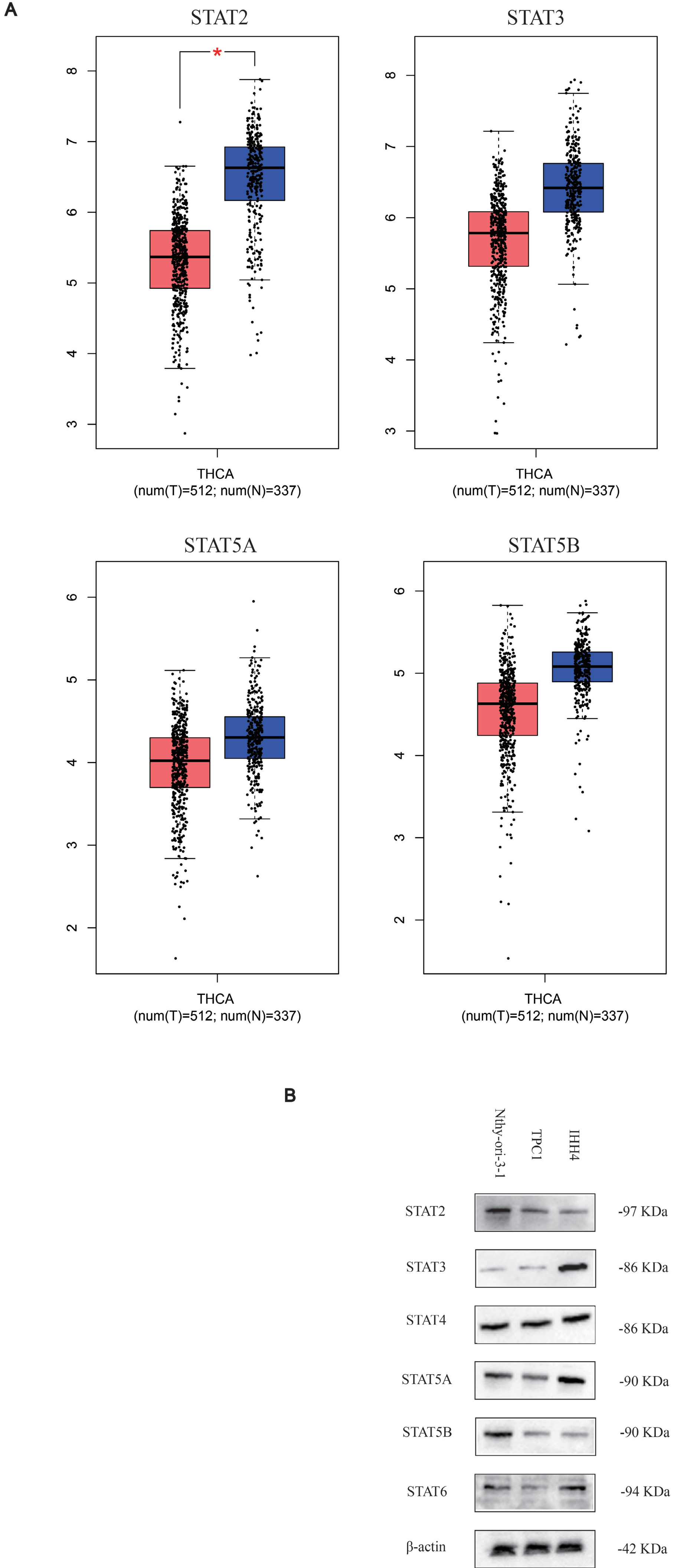
A
STAT2
STAT3
STAT4
STAT5A
STAT5B
STAT6
THCA
(num(T)=512; num(N)=337)
B
Nthy-ori-3-1
TPC1
IHH4
STAT2
-97 KDa
STAT3
-86 KDa
STAT4
-86 KDa
STAT5A
-90 KDa
STAT5B
-90 KDa
STAT6
-94 KDa
β-actin
-42 KDa

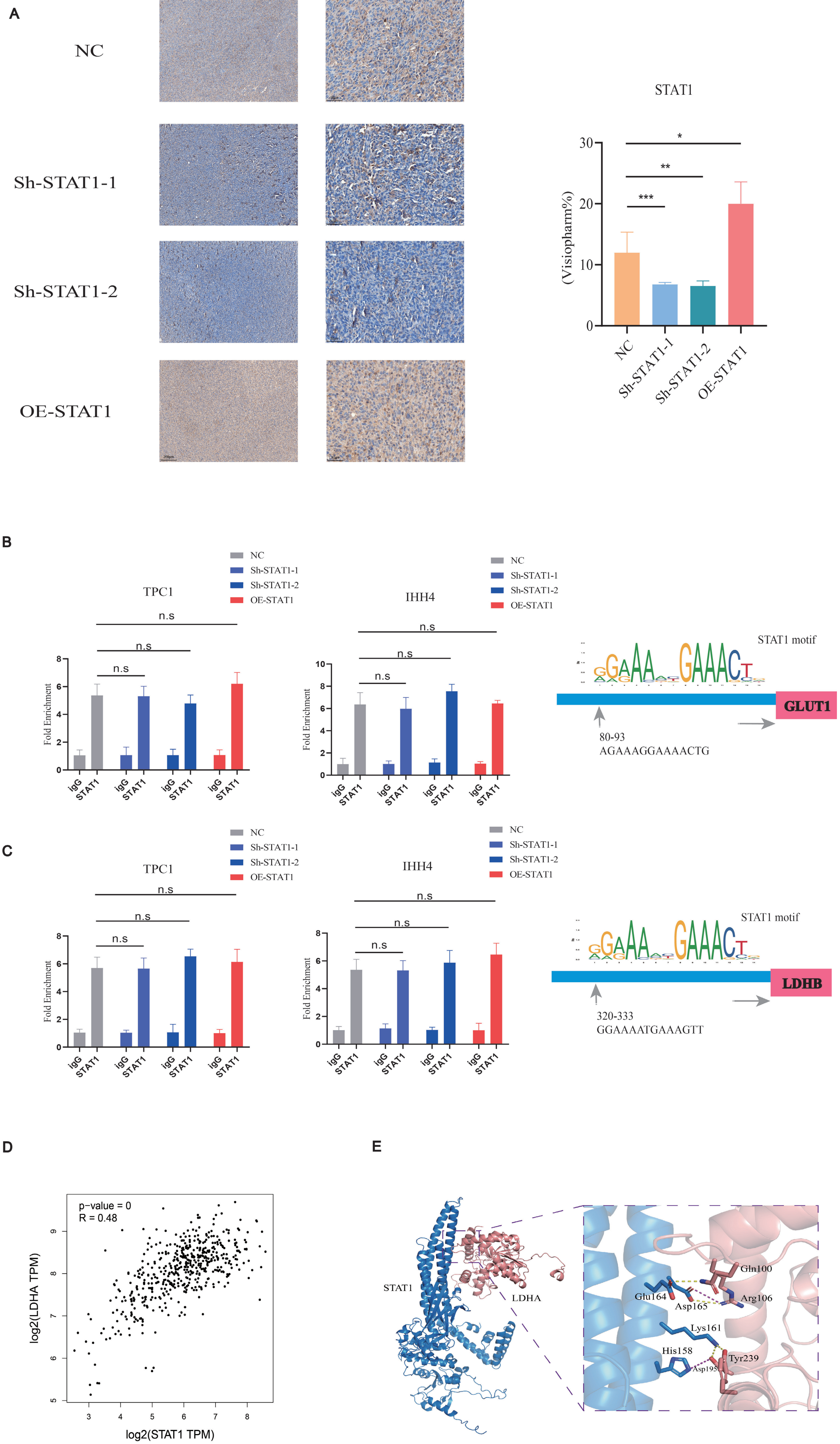
A
NC
Sh-STAT1-1
Sh-STAT1-2
OE-STAT1
STAT1
(Visiopharm%)
30
20
10
0
*
**
***
NC
Sh-STAT1-1
Sh-STAT1-2
OE-STAT1
B
TPC1
NC
Sh-STAT1-1
Sh-STAT1-2
OE-STAT1
n.s
Fold Enrichment
igG
STAT1
IHH4
STAT1 motif
GLUT1
80-93
AGAAAGGAAAACTG
C
TPC1
IHH4
STAT1 motif
LDHB
320-333
GGAAAATGAAAGTT
D
p−value = 0
R = 0.48
log2(LDHA TPM)
log2(STAT1 TPM)
E
STAT1
LDHA
Gln100
Glu164
Asp165
Arg106
Lys161
His158
Asp195
Tyr239
